# Production of Blended Poly(acrylonitrile): Poly(ethylenedioxythiophene):Poly(styrene sulfonate) Electrospun Fibers for Neural Applications

**DOI:** 10.3390/polym15132760

**Published:** 2023-06-21

**Authors:** Fábio F. F. Garrudo, Giulia Filippone, Leonor Resina, João C. Silva, Frederico Barbosa, Luís F. V. Ferreira, Teresa Esteves, Ana Clara Marques, Jorge Morgado, Frederico Castelo Ferreira

**Affiliations:** 1 Instituto de Telecomunicações, Instituto Superior Técnico, Universidade de Lisboa, Avenida Rovisco Pais, 1049-001 Lisboa, Portugal; 2 Department of Bioengineering, Instituto Superior Técnico, Universidade de Lisboa, Avenida Rovisco Pais, 1049-001 Lisboa, Portugaljoao.f.da.silva@tecnico.ulisboa.pt (J.C.S.);; 3 iBB—Institute for Bioengineering and Biosciences, Instituto Superior Técnico, Universidade de Lisboa, Avenida Rovisco Pais, 1049-001 Lisboa, Portugal; 4 Associate Laboratory i4HB—Institute for Health and Bioeconomy, Avenida Rovisco Pais, 1049-001 Lisboa, Portugal; 5 Departament d’Enginyeria Química and Barcelona Research Center for Multiscale Science and Engineering, EEBE, Universitat Politècnica de Catalunya, 08019 Barcelona, Spain; 6 Department of Chemical Engineering, Instituto Superior Técnico, Universidade de Lisboa, Avenida Rovisco Pais, 1049-001 Lisboa, Portugal; 7 CERENA, DEQ, Instituto Superior Técnico, Universidade de Lisboa, Avenida Rovisco Pais, 1049-001 Lisboa, Portugal

**Keywords:** neural tissue engineering, materials science and chemistry, spin-coating, electrochemically active blends, neural stem cells

## Abstract

This study describes, for the first time, the successful incorporation of poly(ethylenedioxythiophene):poly(styrene sulfonate) (PEDOT:PSS) in Poly(acrylonitrile) (PAN) fibers. While electroconductive PEDOT:PSS is extremely challenging to electrospun into fibers. Therefore, PAN, a polymer easy to electrospun, was chosen as a carrier due to its biocompatibility and tunable chemical stability when cross-linked, particularly using strong acids. PAN:PEDOT:PSS blends, prepared from PEDOT:PSS Clevios PH1000, were electrospun into fibers (PH1000) with a diameter of 515 ± 120 nm, which after being thermally annealed (PH1000 24H) and treated with heated sulfuric acid (PH1000 H_2_SO_4_), resulted in fibers with diameters of 437 ± 109 and 940 ± 210 nm, respectively. The fibers obtained over the stepwise process were characterized through infra-red/Raman spectroscopy and cyclic voltammetry. The final fiber meshes showed enhanced electroconductivity (3.2 × 10^−3^ S cm^−1^, four-points-assay). Fiber meshes biocompatibility was evaluated using fibroblasts and neural stem cells (NSCs) following, respectively, the ISO10993 guidelines and standard adhesion/proliferation assay. NSCs cultured on PH1000 H_2_SO_4_ fibers presented normal morphology and high proliferation rates (0.37 day^−1^ vs. 0.16 day^−1^ for culture plate), indicating high biocompatibility for NSCs. Still, the low initial NSC adhesion of 7% calls for improving seeding methodologies. PAN:PEDOT:PSS fibers, here successful produced for the first time, have potential applications in neural tissue engineering and soft electronics.

## 1. Introduction

**Figure 1:**
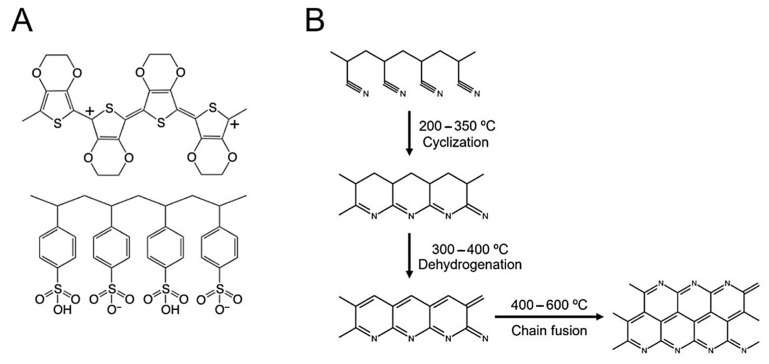
Chemical structure of the polymers used in the design of PAN:PEDOT:PSS fibers. (**A**) Poly(3,4-ethylenedioxythiophene):poly(styrene sulfonate) (PEDOT:PSS); (**B**) Poly(acrylonitrile) (PAN) and respective stabilization/graphitization reactions.

There is an urgent need for the development of electroconductive materials suitable for neural regeneration [1,2]. Electroconductive polymers are a class of smart biomaterials suitable for tissue engineering (TE) applications. One such polymer is poly(3,4-ethylenedioxythiophene):poly(styrene sulfonate) (PEDOT:PSS) (Figure 1A), an electroconductive polymer described in the literature as having high thermal/electrochemical stability, high electroconductivity, and favorable biocompatibility for neural cells [3,4]. The electroconductivity of PEDOT:PSS can be enhanced by pseudo-doping agents (DMSO, ethylene glycol, methanol) [5,6] or treatment with sulfuric acid [7,8]. This last method is considered the most efficient for enhancing the electroconductivity of spin-coated PEDOT:PSS films up to 1000–5000 S cm^−1^ [9]. PEDOT:PSS is mainly available as commercial water dispersions, and thus, processability into relevant TE structures is limited to the production of spin-casted films, coatings, hydrogels, and electrospun fibers made with water soluble carrier-polymers. The works of Lu and colleagues [10] and Yuk and colleagues [11] demonstrate that PEDOT:PSS can be precipitated and redispersed into a new solvent system without losing electrical properties. As such, this work widens the possibility of dispersing PEDOT:PSS into new solvent systems.

PEDOT:PSS has been used in the development of platforms suitable for electrical stimulation, scaffolds for neural TE [12,13,14], and sensors for brain activity [15,16,17]. For example, Alba and colleagues [18], later followed up by Golabchi and colleagues [19] from the same group, studied the application of PEDOT:PSS—multiwalled carbon nanotubes (MWNTs) coating for the development of brain sensors. They found that the PEDOT:PSS-MWNTs coating greatly enhanced the interactions of neural cells with an implanted device, similarly to what was observed for the cell adhesion molecule L1 protein coated electrode by Golabchi and colleagues. While the molecular mechanism is subject to debate, Golabchi and colleagues hypothesize that this phenomenon arises by an improvement in cell adhesion and interaction with the electrode, and reduced neuroinflammation associated with the implanted electrode. This is mediated by a more bioactive surface, either due to its surface charge or immobilized adhesion proteins, which lead to reduced tissue rejection after implantation and improves the recording ability and usage time of the electrodes.

Electrospinning is a technique that allows the production of nanometric-sized fibers. The obtained fibers have many advantages for TE applications, including (a) structural resemblance to native extracellular matrix, (b) high surface area for cell adhesion, and (c) ability for functionalization (e.g., coating/immobilization) with bioactive motifs and/or drugs. PEDOT:PSS cannot be electrospun alone due to unfavorable chain entanglement during the solvent evaporation stage [20], and thus, requires a carrier polymer [21] or a cross-linker such as magnesium ion (Mg^2+^) [22]. As such, several electroactive composites and blends have been developed for the electrospinning of scaffolds for neural TE applications [23,24,25]. However, the number of PEDOT:PSS-based blends suitable for electrospinning remains limited due to three mains reasons: (a) most commercially available PEDOT:PSS formulations are aqueous dispersions, which limits the number of PEDOT:PSS carrier polymers to those that are water soluble [9]; and (b) PEDOT:PSS requires cross-linking to avoid its dispersion in water upon immersion, which hampers the electroconductivity and integrity of the blend [26,27], and (c) PEDOT:PSS enhancement of electroconductivity and stabilization in aqueous systems often requires the use of post-processing treatments, which conditions, such as heated sulfuric acid, are not always compatible with chemical stability of the carrier polymer.

Poly(acrylonitrile) (PAN) is a promising polymer for biomedical applications, and a candidate to be a carrier polymer for PEDOT:PSS. This material presents numerous advantages, including good mechanical properties, chemical stability, and ease of industrial production [28]. PAN can also be cross-linked to better resist the necessary acidic treatments to enhance the electroconductivity of PEDOT:PSS. This process begins by heating PAN at high temperatures (200–350 °C), which promotes its cyanide groups to react with other cyanide groups in the vicinity, leading to the formation of a chain of fused rings with the formation of a π-conjugated chain involving the nitrogen atoms (cyclization), as shown in Figure 1B.

A dehydrogenation process can then take place at 300–400 °C, leading to highly π-delocalized system (stabilized PAN fiber) and, at slightly higher temperatures, there starts to occur the lateral fusion of chains (cross-linking). Thermal treatment at higher temperatures (above 600 °C) leads to graphitization with the formation of PAN-derived carbon fibers. The process can be performed under both inert [29,30] or regular [31,32,33] atmosphere. The extension of graphitization can be increased by the use of high temperatures (1000–1200 °C), long times of reaction, and adequate cyclization/dehydrogenation steps pre-graphitization. This leads to an increase in polycyclic structures in PAN, leading to posterior formation of more graphite and improvements in the obtained electroconductivity [34]. Overall, pristine and graphitized PAN are biocompatible and have been used in the production of scaffolds for culture and differentiation of ESCs [35], MSCs [36], and NSCs under electrical stimulation [37], and also, to develop nerve conduits [38].

In this work, we propose the development of new electroconductive blend to produce electrospun fibers of PEDOT:PSS and PAN. There are no reports in the literature for electroconductive PAN:PEDOT:PSS blend electrospun fibers, although some reports describe similar electroconductive fibers using different strategies [39,40,41,42]. The strategy employed for their production is based on the recent work of Yuk and colleagues [11], who successfully created highly concentrated PEDOT:PSS solutions (1–10%) suitable for the 3D printing (5–7%) of highly electroconductive devices. PAN was chosen as the carrier polymer of PEDOT:PSS because, when cross-linked, it resists the necessary acid treatment to enhance the electroconductivity of PAN:PEDOT:PSS fibers before the biological tests. For the first time, we created electroconductive PAN:PEDOT:PSS blend electrospun fibers, evaluated their physico–chemical properties, and tested their suitability for neural TE applications. Spin-coated films of PEDOT:PSS were also prepared in parallel for the easy study of the influence of processing conditions on PEDOT:PSS. We believe that the results described herein will provide a steppingstone for the generation of a new PEDOT:PSS-based material for neural TE and soft electronics applications.

## 2. Materials and Methods

### 2.1. Materials

Medical glue (silastic^®^ medical adhesive silicone type A) was obtained from Biesterfeld Spezialchemie Ibérica, S.L.U. (Barcelona, Spain). The primary antibody Anti-(Sex determining region Y)-box 2 (Anti-SOX2) (rabbit) (orb607714) was purchased from Byorbyt (Cambridge, UK). Ultra-low attachment 24-well plates (flat bottom) were obtained from Corning (Corning, NY, USA). Dimethylformamide (DMF) was purchased from Fisher Scientific. PEDOT:PSS dispersions Clevios^TM^ PH 1000 (PEDOT:PSS ratio of 1:2.5, solids content 1.0–1.3%) and Clevios^TM^ P VP AI 4083 (PEDOT:PSS ratio of 1:6, solids content 1.3–1.7%) were purchased from Heraeus (Hanau, Germany). Sulfuric acid (95.0–97.0%) was purchased from Honeywell (Charlotte, NC, USA). 3-(4,5-dimethylthiazol-2-yl)-2,5-diphenyltetrazolium bromide (MTT), dimethyl sulfoxide ≥99.5% (DMSO), glucose, human recombinant insulin, 2-(4-amidinophenyl)-6-indolecarbamidine dihydrochloride (DAPI), paraformaldehyde (PFA) (crystalline), phalloidin–tetramethylrhodamine B isothiocyanate and poly(L-ornithine hydrobromide) were purchased from Merck-Millipore (Burlington, MA, USA). Human recombinant epidermal growth factor (EGF) and human recombinant fibroblast growth factor 2 (FGF-2) were purchased from Peprotech (London, UK). PAN (MW 200,000) was purchased from Polysciences Inc (Warrington, PA, USA). The primary antibody Anti-Nestin (Anti-NES) (mouse) (MAB1259) was purchased from R&D systems (Minneapolis, MN, USA). Dulbecco’s phosphate buffer saline (PBS), Dulbecco’s Modified Eagle’s Medium (DMEM:F12 + glutamax (1×), Fetal Bovine Serum (FBS), Pen/Strep mixture (penicillin 10,000 units mL^−1^, streptomycin 10,000 µg mL^−1^), Antibiotic–Antimycotic mixture (penicillin 10,000 units mL^−1^, streptomycin 10,000 µg mL^−1^, and Amphotericin B 25 µg mL^−1^) (Anti-Anti), N2-supplement (100×), B27-supplement (50×), Alamar Blue^®^ cell viability reagent, calcein AM, ethidium bromide, triton-x-100 (Surfact-Amps^®^, 10% in water), secondary antibodies Alexa 488 anti-mouse IgG and Alexa 546 anti-rabbit IgG were purchased from Thermofisher (Waltham, MA, USA). Frozen stocks of ReNCell-VM (SCC008) (Merck Millipore, Burlington, MA, USA) (Passage 8) and L-929 mouse fibroblasts (ATCC CCL1) (Passage 6) were used in these studies.

### 2.2. PAN:PEDOT:PSS Electrospun Fiber Production

#### 2.2.1. Solution Preparation

To 10 mL of commercially available PEDOT:PSS dispersions in water Clevios^TM^ PH1000 or Clevios^TM^ AI4083, 1 mL of DMSO was added. The obtained dispersions were left to evaporate overnight at 60 °C in a Heratherm OMS60 convection oven (Thermo Scientific, Waltham, MA, USA). The obtained PEDOT:PSS pellets were then subjected to 3 consecutive cycles of heat-treatment at 130 °C for 30 min and mechanical crushing. An amount of 10 mL of DMF:DMSO (9:1) was added to the obtained solid. Complete dispersion of PEDOT:PSS pellets was achieved by constant agitation (300–350 rpm) using magnets for at least 2 days (Clevios^TM^ AI4083) or 5 days (Clevios^TM^ PH1000).

PAN and PAN:PEDOT:PSS solutions were prepared by adding 1 g of PAN to 10 mL of DMF or to 10 mL of PEDOT:PSS dispersion in DMF:DMSO (9:1). Solutions were left to agitate for 1–3 h at 85 °C. If not used promptly, PAN:PEDOT:PSS solutions were stored at 4 °C for up to 24 h.

#### 2.2.2. Electrospinning

PAN and/or PAN:PEDOT:PSS solutions were electrospun using a spinneret system (MECC, Ogori, Fukuoka, Japan) and a 21G needle (0.51 mm of internal diameter) under the following conditions: voltage of 20 kV, flow rate at 1.0 mL h^−1^, distance from needle to collector of 25 cm, temperature of 25 °C, relative humidity of 50%. The electrospinning setup also included a high voltage power source (Series EL, Model ES/EL40P01, XP Glassman High Voltage, Inc, High Bridge, NJ, USA), a syringe pump (Model NE-1000, New Era Pump Systems, New Farmingdale, NY, USA) and a teflon tube (Henke Sass Wolf, Dudley, MA, USA). Samples were collected on an aluminum foil-coated static copper collector. An air-blower was used to force fiber deposition on the collector.

Electrospun fiber samples were named according to their composition, in particular to the source of PEDOT:PSS used in their composition. They are: pristine PAN fibers—“Samples PAN”; PAN:PEDOT:PSS fibers made with PEDOT:PSS dispersion Clevios^TM^ P VP AI 4083—“Samples AI4083”; and PAN:PEDOT:PSS fibers made with PEDOT:PSS dispersion Clevios^TM^ PH 1000—“Samples PH1000”.

#### 2.2.3. Post-Processing

The obtained electrospun fibers were subjected to thermal treatment at a steady temperature (210 °C) in a drying oven (Model E 28, Binder GmbH, Tuttlingen, Germany) for 1, 5, 10, and 24 h under ambient pressure. Samples were then collected and let to cool down to RT before use.

PAN and PAN:PEDOT:PSS fibers were subjected to a final treatment with heated sulfuric acid. Briefly, fibers were incubated for 24 h with sulfuric acid for complete soaking. The collected fibers, soaked in sulfuric acid, were then uniformly heated for 30 min at 130 °C, cooled to RT and thoroughly washed with distilled water. Other incubation conditions are also reported as part of the optimization steps to enhance the electroconductivity of PAN:PEDOT:PSS fibers, including soaking with sulfuric acid for 1 h, and heat treatments at 100 °C or 150 °C.

### 2.3. Production and Characterization of PEDOT:PSS Spin-Coated Films

PEDOT:PSS films were produced by spin-coating (1200 rpm for 12 s, followed by 1800 rpm for 45 s) of PEDOT:PSS dispersions with 10% DMSO on pre-cleaned glass/quartz coverslips. The samples were dried at 125 °C. Further processing was used to match the processing conditions of PAN:PEDOT:PSS fibers, as described in Section 2.2.3.

### 2.4. Sample Physico–Chemical Characterization

#### 2.4.1. Scanning Electron Microscopy (SEM)

The morphology of the electrospun fiber mats was evaluated using SEM (Hitachi S-2400, Chiyoda, Tokyo, Japan; and FEG-SEM JEOL JSM7001F, Akishima, Tokyo, Japan) after coating with a thin layer of gold/palladium. The average diameter of the electrospun fiber samples was determined for 50–100 individual fibers using NIH ImageJ software (National Institute of Health, Bethesda, MD, USA). The diameters obtained were averaged and the histograms were plotted using Microsoft Excel.

#### 2.4.2. UV-Visible Spectroscopy (UV/Vis)

The spectra of the spin-coated PEDOT:PSS films, prepared on quartz coverslips, were obtained using a V-730 UV-Visible Spectrophotometer (Jasco, Easton, MD, USA) in the 200–1100 nm range (resolution of 0.2 nm). The obtained spectra were normalized to the maximum peak found in the 225–235 nm region.

#### 2.4.3. Attenuated Total Reflectance—Fourier-Transform Infrared Spectroscopy (ATR—FTIR)

The obtained PAN and PAN:PEDOT:PSS fibers were analyzed by ATR—FTIR using a Spectrum Two FT-IR Spectrometer (Perkin-Elmer, Waltham, MA, USA), equipped with a Pike Technologies MIRacle^®^ ATR accessory (Fitchburg, WI, USA). Transmittance spectra were obtained from 400 to 4000 cm^−1^ (resolution of 4 cm^−1^, accumulation of 8 scans) at room temperature and an automatic baseline correction treatment was applied using the acquisition software. The obtained spectra were normalized to the maximum peak obtained.

#### 2.4.4. Micro-Raman Spectroscopy

The spectra of PAN fibers, PAN:PEDOT:PSS fibers, and PEDOT:PSS spin-coated films were obtained using a Renishaw In-Via confocal Raman microscope (Renishaw, Wotton-under-Edge, England, UK), using a 532 nm excitation and 10× objective (resolution of 1 cm^−1^), and Renishaw software Wire 4. The obtained spectra were normalized to the maximum peak found in the 1200–1700 cm^−1^ region.

#### 2.4.5. Four-Probe Electroconductivity

PAN fibers, PAN:PEDOT:PSS fiber mats and PEDOT:PSS spin-coated films were analyzed by the four-probe method to evaluate electroconductivity. Four 50 nm thick gold stripes were deposited by thermal evaporation (Edwards Coating System E 306A (Edwards, Irvine, CA, USA)) to improve the electrical contact between the samples and the measurement equipment. The electrical resistance of 5 different films was measured by the 4-point probe method, using a current source Keithley 2400 DC power source (Keithley Instruments, Cleveland, OH, USA) and a multimeter Agilent 34401A Multimeter (Agilent Technologies, Santa Clara, CA, USA). Finally, the thicknesses of the fiber mats and spin coated films, used to calculate the electroconductivity, were measured, respectively, using a caliper and a Bruker’s Dektak^®^ 3.21 profilometer (Billerica, MA, USA).

#### 2.4.6. Cyclic Voltammetry

Cyclic voltammetry scans were run for PAN fibers and PAN:PEDOT:PSS fibers using working and auxiliary carbon screen printed electrodes (SPEs) and an Ag|AgCl reference electrode (Metrohm DropSens, Herisau, Switzerland), connected to a potentiostat (400B Electrochemical Analyzer, CH Instruments, Austin, TX, USA). Fiber samples were deposited on the working electrode surface. The electrodes were dipped in 2.5 mL of degassed PBS 0.01 M, pH 7.4, as electrolytic medium. Cycles were run in a potential window of −0.7 V to +0.7 V, with scan rates ranging from 0.01 V s^−1^ to 0.2 V s^−1^.

#### 2.4.7. Mechanical Properties

The mechanical properties of PAN and PAN:PEDOT:PSS fibers were analyzed using a texture analyzer (TA.XT ExpressC Texture Analyzer, Stable Micro Systems, Godalming, UK) equipped with 50 N load cell and tensile grips. Specimens were cut into rectangular strips (40 mm × 10 mm, *n* = 5 different samples) and the crosshead speed was set constant at 10 mm min^−1^ during the uniaxial test. Young’s modulus was calculated from the 0–15% strain linear region in the stress−strain curve. The ultimate tensile strength (UTS) and the maximum extension were measured from the highest peak of the stress−strain curve.

### 2.5. ISO-10993 Biocompatibility Assays

Cytotoxicity tests were performed following the recommendations of ISO 10993-5 and 10993-12 guidelines. Briefly, mouse fibroblast cell line L-929 (Passage 10) were cultured in Dulbecco’s modified eagle’s medium (DMEM + 10% FBS + 1% Anti-Anti) at 37 °C and 5% CO_2_ until confluence before passage to 24-well plates at 150,000 cells cm^−2^ (direct contact) or 80,000 cells cm^−2^ (indirect contact). PAN fibers, PAN:PEDOT:PSS fibers, and latex (positive control) were sterilized with UV (1.5 h per sample side) before further processing. Samples were then incubated in medium composed of DMEM + 10% FBS + 1% anti-anti for 24 h (6 cm^2^ mL^−1^) for lixiviate extraction (indirect contact). For direct contact, each of the samples were placed on top of the cell’s monolayer for 24 h before image analysis on an optical microscope (Leica microsystems CMS GmbH, Wülfrath, Germany). For the indirect contact assay, the media of L-929 cells were switched to media containing lixiviates from processing materials and the cells were left to incubate for 24 h, after which cell viability was quantified using MTT assay. Briefly, after PBS washing, cells were incubated with MTT solution (1 mg mL^−1^) for 2 h at 37 °C. The resulting formazan salt was then dissolved using 0.1 M HCl in isopropanol and absorbance was quantified at 570 nm. Relative cell viability was calculated using the negative control as the reference.

### 2.6. ReNCell-VM Proliferation Assay

#### 2.6.1. ReNCell-VM Maintenance

ReNCell-VM (NSCs immortalized after transduction of *c-myc*) were used as a cell model for neural applications [43,44]. They were grown on poly(L-ornithine) (overnight—20 µg mL^−1^) and laminin (2 h—10 µg mL^−1^) coated plates in supplemented N2 medium at 37 °C and 5% CO_2_, as recommended [45,46]. N2 medium is composed of DMEM/F12 with N2 supplement (1:100), glucose (1.6 g mL^−1^), insulin (20 µg mL^−1^), and pen-strep (1:100). N2 medium was then supplemented with EGF (20 ng mL^−1^), FGF-2 (20 ng mL^−1^), and B27 (20 µL mL^−1^).

#### 2.6.2. Proliferation Assay

Different electrospun samples, previously immobilized on glass coverslips with medical glue, were first UV sterilized for 1.5 h per each sample side and treated with 1% Anti-Anti solution in PBS for 3 h. The samples were then coated with poly(L-ornithine) (overnight—20 µg mL^−1^) and laminin (2 h—10 µg mL^−1^) before being seeded with ReNCell-VM (Passage 10) at 50,000 cells cm^−2^. Supplemented N2 medium was added 1 h after seeding (37 °C and 5% CO_2_) to promote initial cell attachment. Medium was exchanged at day 1, when cell adhesion was calculated, at day 2 and then every 2 days thereafter until the end of the experiment (day 7). Cellular metabolic activity was assessed using Alamar Blue^®^ by measuring fluorescence at 590 nm (excitation at 560 nm) at days 1, 4, and 7. Equivalent cell number was determined using a calibration curve. Cell adhesion (Equation (1)), growth rate, (Equation (2)) and duplication time (Equation (3)) were calculated using the following formulae [20,46]:

Cell adhesion at day 1 (%) = (cells at day 1)/(seeded cells) × 100(1)

Growth rate (h^−1^) = [ln(cells day 6) − ln(cells day 2)]/(total time) (2)

Doubling time (h) = Ln(2)/Growth rate (h^−1^) (3)

where total time are the hours between sample taken at day 7 and day 1.

#### 2.6.3. Morphology and Marker Analysis

At the end of the proliferation assay (day 7), cells were washed once with PBS and then incubated with a staining solution composed of calcein (4 µM), ethidium bromide (2 µM mL^−1^), and glucose (0.056 M) in PBS, for 20 min at 37 °C. Cells were then washed, kept in PBS supplemented with glucose (0.056 M), and imaged using an optical microscope (Leica microsystems CMS GmbH, Wülfrath, Germany) equipped with a fluorescent lamp.

Cell samples were fixed in PFA 4% for 10 min, washed with PBS twice and then permeabilized with staining solution (FBS 5% and Triton-X-100 0.1% in PBS) for 15 min at RT. Cells were incubated with the primary antibodies Anti-NES (1:250) and Anti-SOX2 (1:100) in staining solution, overnight at 4 °C. Next, cells were incubated with the secondary antibodies Alexa 488 IgG Anti-Mouse (1:250) and Alexa 546 IgG Anti-Rabbit (1:250), for 2 h at RT. Counter-staining was performed with DAPI (1 mg mL^−1^). Samples were then washed with PBS twice and kept in PBS until imaging.

### 2.7. Statistical Analysis

Raman data were smoothed on OriginPro 8 SR0 software (OriginLab corporation, Northampton, MA, USA) using the Savitzky–Golay method (200 points of window, polynomial order of 5). Data are presented as mean values ± standard deviations (std). Statistical analysis was performed using Microsoft Excel (Microsoft, Redmond, WA, USA): significant differences between groups were determined using ANOVA test, followed by post hoc analysis and Bonferroni correction. A *p* < 0.05 was considered statistically significant. When found, these differences were indicated in the respective figures/tables.

## 3. Results and Discussion

PAN and PAN:PEDOT:PSS fibers were electrospun to create electroconductive scaffolds for neural applications. PEDOT:PSS, composed of the electrically conductive component PEDOT and its doping agent PSS, was re-dispersed in DMF:DMSO (9:1) following a modified protocol from Lu and colleagues [10]. The electroconductivity of PEDOT:PSS can be greatly enhanced through sulfuric acid and heat treatment [9], but most polymers used as carrier in electrospinning are either easily degraded by sulfuric acid for being not thermostable and/or electroneutral, impairing fiber post-production treatments or final electroconductivities of the blend. PAN is an easily electrospun polymer that can be annealed at high temperatures into a poly(heteroaromatic) electroconductive compound and with high resistance to acid treatment. We hypothesized that electroconductive PAN:PEDOT:PSS fibers can be created by harnessing the thermally annealed PAN [47] in order to obtain acid-resistant fibers. After this, electroconductivity can be increased through sulfuric acid treatment by affecting the PEDOT:PSS component.

### 3.1. Diameter of PAN and PAN:PEDOT:PSS Fibers

**Figure 2:**
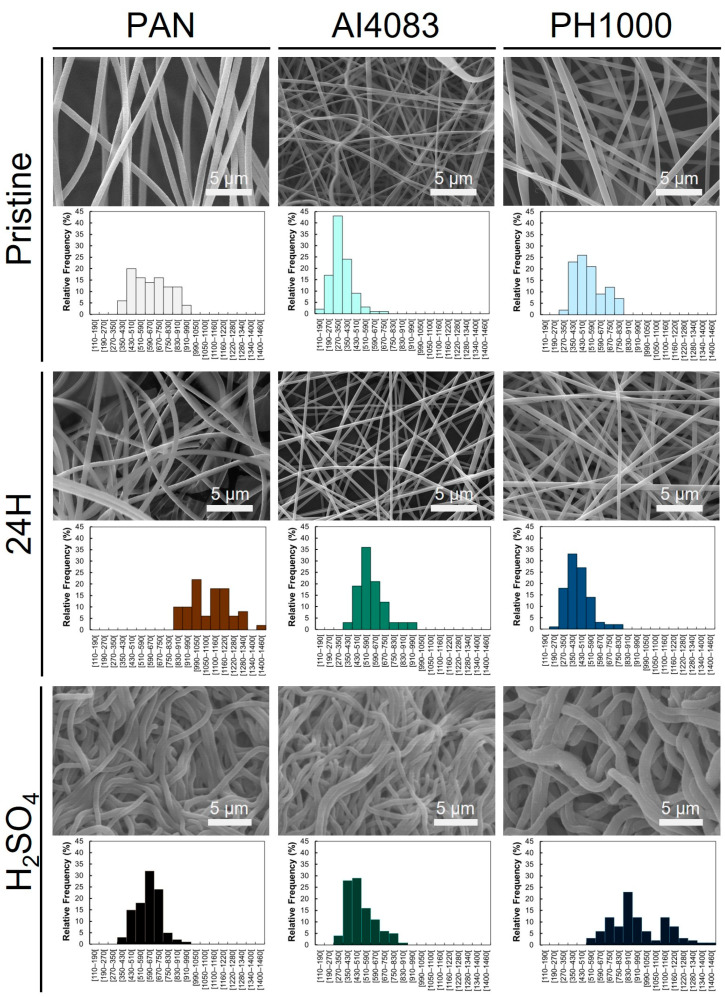
SEM images and respective size distribution histograms for the main fibers obtained in this study.

When 10% PAN fibers are heat-treated, cyclization followed by dehydrogenation occurs, leading to the formation of a π-system that can stand electrical transport. The extent of this change can be accompanied by observable color changes of PAN fibers from white (starting fibers) to light brown (1 h and 5 h samples) and finally to dark brown (10 h and 24 h samples). SEM images (Figure 2 and Figure S1) and respective fiber diameter analysis (Table 1 and Table S1) of samples collected at different timepoints show that thermal annealing does not alter the visual aspect of the obtained fibers.

The obtained PAN fibers were smooth, homogeneous, and bead-free. Untreated PAN fibers (PAN) had an average fiber diameter of 640 ± 159 nm (*n* = 50). Fiber diameter changed when the thermal annealing at 210 °C was performed for 1 h (PAN 1H—759 ± 98 nm, *n* = 50), 5 h (PAN 5H—654 ± 135 nm, *n* = 50), 10 h (PAN 10H—703 ± 85 nm, *n* = 50), and 24 h (PAN 24H—1100 ± 135 nm, *n* = 50). When sample PAN 24H was incubated with heated sulfuric acid (PAN H_2_SO_4_), the obtained fiber diameter was 612 ± 107 nm (*n* = 100), which is statistically different from the values obtained for PAN, PAN 10H, and PAN 24H (without acid treatment). Further observed statistical differences found are summarized in Table 1 and Table S1. Even though thermal annealing for 24 h greatly affected the obtained fiber diameter, no specific trend on fiber diameter change was observed. Visually (Figure 2 and Figure S1), the sequential thermal annealing and sulfuric acid treatment did not affect fiber morphology significantly. Finally, sulfuric acid treatment induced changes in fiber compaction. This observation when associated with changes in fiber diameter suggests a change in fiber mat porosity.

The obtained fibers of PAN:PEDOT:PSS AI4083 pristine (AI4083—356 ± 104 nm, *n* = 100) and PAN:PEDOT:PSS PH1000 pristine (PH1000—515 ± 120 nm, *n* = 100) were also smooth, homogeneous, and bead-free (Figure 2). The obtained diameters were not statistically different from each other, indicating the composition of PEDOT:PSS does not affect the morphology of the fibers. Thermal annealing of the fibers induced slight changes on fiber diameter for both PAN:PEDOT:PSS AI4083 24 h (AI4083 24H—592 ± 117 nm, *n* = 100) and PAN:PEDOT:PSS PH1000 24 h (PH1000 24H—437 ± 109 nm, *n* = 100). However, no statistically significant differences between these fibers and their pristine counterparts were found. Incubation with heated sulfuric acid led to morphological changes in samples PAN:PEDOT:PSS AI4083 24 h + H_2_SO_4_ (AI4083 H_2_SO_4_—507 ± 125 nm, *n* = 100) and PAN:PEDOT:PSS PH1000 24 h + H_2_SO_4_ (PH1000 H_2_SO_4_—940 ± 210 nm, *n* = 100). The increase in fiber diameter of sample PH1000 H_2_SO_4_ is statistically significant with respect to samples PH1000 and PH1000 24H. Moreover, we also observed statistically significant differences between PH1000 H_2_SO_4_ and samples AI4083 H_2_SO_4_ and PAN H_2_SO_4_. The commercial formulations of PEDOT:PSS Clevios^TM^ PH 1000 and Clevios^TM^ P VP AI 4083 have different PEDOT to PSS ratios. As such, the relative amount of PEDOT present in the PH1000 H_2_SO_4_ sample is higher than in the AI4083 H_2_SO_4_ sample. This suggests that the increase in fiber diameter, observed for sample PH1000 H_2_SO_4_, is related to a higher amount of PEDOT (or to a lower content of PSS). This is a quite unexpected result. PEDOT-rich domains are hydrophobic and PSS-rich domains are hydrophilic [35] and it has also been reported that sulfuric acid treatment induces the removal of PSS from the blend samples and increased crystallinity of the PEDOT-rich domains [48]. Kim and colleagues associate this treatment with an increase in the surface hydrophobic character (pristine PEDOT: PSS films = 26.53°, equivalent film treated with 15 M H_2_SO_4_/DMSO = 38.50°) [49]. Therefore, the sulfuric acid treatment should instead lead to a reduction of the fiber diameter, this being expected to be more pronounced for the fibers based on the PEDOT:PSS blend with higher PSS content (AI4083). As mentioned above, the diameter of the PAN fibers instead undergoes a reduction upon treatment with sulfuric acid, wherein the diameter of PAN 24H fibers decrease from 1100 ± 135 nm (*n* = 50) to 612 ± 107 nm (*n* = 100) when incubated with heated sulfuric acid. This reduction is also unexpected as, should there be a protonation of the imine nitrogens of thermally annealed PAN, we would anticipate an expansion instead. It is hard to find an explanation for these observations. Further studies are necessary to fully understand this phenomenon.

Our results suggest that thermal annealing of PAN and PAN:PEDOT:PSS fibers had a mixed impact on fiber diameter. We were curious whether these changes could also be associated with the chemical reactions involved in the cyclization/dehydrogenation performed to the fibers. As described in the literature, PAN fibers (control fibers) are typically turned electroconductive after four different sequential reactions, which can affect the obtained fiber diameter due to variable mass loss: cyclization; oxidation/dehydrogenation; carbonization; and graphitization (Figure 1B). Cyclization is started by heating PAN at high temperatures (200–350 °C), which promotes the reaction between adjacent cyanide groups and leads to the formation of interconnected aromatic rings along PAN’s chain. This reaction can be conducted in the absence or presence of oxygen, but the involved molecular mechanism will be different: in an inert atmosphere (no oxygen), the mechanism is preferentially radical dependent [29,30]; in a normal atmosphere, oxygen oxidizes PAN and promotes dehydrogenation and subsequent oxidation until cyclization is complete [31,32,33]. Carbonization usually follows at temperatures ranging from 750 to 1200 °C, to remove any moisture or reaction sub-products formed. When prolonged, graphitization occurs, leading to the orientation of the carbon layers in PAN fibers and formation of conductive graphite ribbons [50]. Alarifi and colleagues report that fibers diameter can decrease with increasing carbonization (750–950 °C) time due to mass loss [30]. In our work, we are using lower temperatures and a longer thermal annealing step (24 h). We predict that mass loss is also likely to occur and impact fiber diameter similarly [31,51,52], especially through the loss of hydrogen, despite the fact that the annealing temperature (210 °C) is slightly lower than the reported temperature for the dehydrogenation to take place (300–400 °C) (Figure 1). We found that PAN fiber diameter remained relatively constant through the thermal annealing up to 10 h, and an increase in fiber diameter is observed only after 24 h. We note that previous reports on the characterization of PAN fibers with thermal treatment above 300 °C show a reduction of diameter up to 1250 °C, while there is an increase of density up to 900 °C [53]. The thermal treatment of the fibers based on the PAN:PEDOT:PSS blends led to divergent results: while the diameter of samples AI4083 24H is higher, the diameter of sample PH1000 24H is lower than that of as-prepared corresponding fibers. This indicates that mass loss, which is small, is not the main driver for the changes observed in fiber diameter.

### 3.2. Evaluation of the Structural Effect of Thermal Annealing on PAN and PAN:PEDOT:PSS

**Figure 3:**
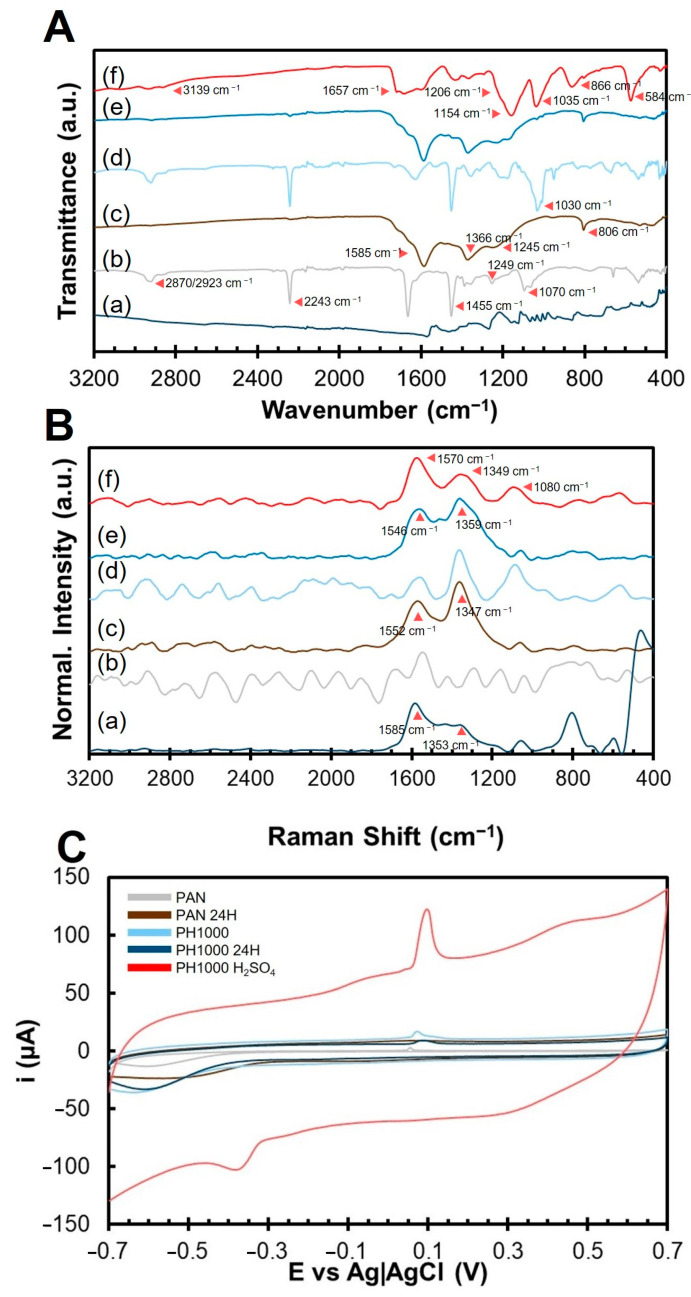
Physico–chemical properties of the obtained PAN:PEDOT:PSS fibers in this study: (**A**) FTIR spectra (normalized to the peak with minimum transmission); (**B**) Raman spectra (normalized to the maximum peak found in the 1200–1700 cm^−1^ region), (**C**) Cyclic voltammetry. Samples analyzed include (a) PEDOT:PSS pellet, and fibers of (b) PAN, (c) PAN 24H, (d) PH1000, (e) PH1000 24H, and (f) PH1000 H_2_SO_4_.

In our work, PAN thermal annealing was performed under a normal atmosphere. The presence of oxygen is positive for the annealing of PAN, as it can act as a catalyst for PAN dehydrogenation [31,33]. To study this chemical process, PAN structural changes, throughout thermal annealing and after treatment with heated sulfuric acid, were monitored through FTIR (Figure 3A and Figure S2).

The FTIR spectrum of PAN fibers (Figure 3A, spectrum (b); Figure S2, sample (a)) exhibits the main characteristic peaks of PAN: the CH_2_ bending peaks at 1070 cm^−1^, 1249 cm^−1^, and 1455 cm^−1^, the double peaks at 2870 cm^−1^ and 2923 cm^−1^ for CH_2_ bonds stretching, and the characteristic nitrile (C≡N) peak at 2243 cm^−1^ [28]. An extra peak at 1666 cm^−1^ can be associated with potential contaminant carbonyl/carboxyl groups in PAN due to partial hydrolysis of nitrile groups due to atmospheric humidity.

The spectra of the different PAN samples change with thermal annealing progression, and the majority of the peaks can be identified in the literature [28,54,55,56]. Changes in PAN’s FTIR spectrum appear after 1 h of thermal annealing (Figure S2A, spectrum (b)). The peak at 1666 cm^−1^ (C=O), attributed to potential contaminant carbonyl/carboxyl groups in PAN, disappears completely. The changes in FTIR spectrum suggest that after 1 h, the cyclization reaction started, which is supported by the appearance of peak at 1585 cm^−1^, associated with C=N and C=C groups. After 5 h of thermal annealing (Figure S2A, spectrum (c)), the new peaks at 806 cm^−1^ (C=C–H), 1366 cm^−1^ (CH—methine group) and 1585 cm^−1^ (C=N and C=C), are clearly visible [50,57]. Other changes observed include a decrease in the intensity of peaks 1452 cm^−1^, 1245 cm^−1^, and 1070 cm^−1^ (CH_2_ groups). This change seems to indicate that the PAN dehydrogenation, expected to occur above ca. 300 °C, occurs upon prolonged heating at 210 °C. After 10 h of thermal annealing (Figure S2A, spectrum (d)) no major changes were observed in the peaks present. The data indicates an increase in the intensity of peaks 1366 cm^−1^ (CH—methine group) and 1585 cm^−1^ (C=N and C=C), suggesting a progression of cyclization and dehydrogenation with the annealing time. After 24 h of thermal annealing (Figure 3A, spectrum (c); Figure S2A, spectrum (e)), 4 main peaks are visible: peaks 806 cm^−1^ (C=C–H), 1366 cm^−1^ (CH—methine group), and 1585 cm^−1^ (C=N and C=C), associated with aromatic rings formed by the combination of cyclization and dehydrogenation, and a peak at 1245 cm^−1^ (CH_2_ bonds), suggesting the presence of residual saturated carbons. At this stage, the majority of peaks characteristic of pristine PAN lost intensity or disappeared, including the double peaks at 2870 cm^−1^ and 2923 cm^−1^ (CH_2_ bonds), the peak at 2243 cm^−1^ (C≡N), and peaks 1452 cm^−1^ and 1070 cm^−1^ (CH_2_ groups). After treatment of sample PAN 24H with heated sulfuric acid (Figure S2A, spectrum (f)), FTIR spectrum changes significantly. Peaks at 806 cm^−1^, 1366 cm^−1^, and 1585 cm^−1^, all associated with aromatic rings, are still present. These now coexist with other peaks, including the broad band centered at 3193 cm^−1^ (O–H), at 1657 cm^−1^ (C=O), and at 1206 cm^−1^, 1096 cm^−1^, 1048 cm^−1^, and 584 cm^−1^ (CH_2_). These results suggest partial hydrolysis of thermally annealed PAN with heated sulfuric acid.

Changes in the spectra of PAN:PEDOT:PSS fibers AI4083 and PH1000 follow a similar trend regarding changes in PAN-characteristic peaks (Figure S2B and Figure S2C respectively). This indicates that incorporation of PEDOT:PSS does not affect PAN structurally nor the progression of the cyclization and dehydrogenation reactions with thermal annealing at 210 °C. Sample PH1000 (Figure 3A, spectrum (d)) presents the characteristic peaks of PAN and a very intense peak at 1030 cm^−1^ potentially attributed to the sulfoxide group of PSS. The intensity of peak 1030 cm^−1^ also decreases with the progression of thermal annealing (Figure S2C), suggesting that the sulfonate group is degraded during the thermal annealing. Sample PH1000 24H (Figure 3A, spectrum (e)) presents the characteristic peaks of PAN 24H, indicating the presence of polycyclic and stabilized PAN. The FTIR spectra for the PAN fibers heat-treated for 10 h and 24 h (Figure 3A and Figure S2) are similar to that of stabilized PAN fibers reported by Li and colleagues [58]. It is relevant to mention that upon treatment of the 24 h annealed samples of PAN:PEDOT:PSS (AI4083 H_2_SO_4_ and PH1000 H_2_SO_4_) (Figure S2(Bl,Cr)), a peak at ca. 1030 cm^−1^ appears, which is likely due to the presence of HSO_4_^−^ species.

The effect of sulfuric acid treatment of sample PH1000 24H, aiming to improve its electroconductivity, was monitored using FTIR (Figure S3). The goal of this step was to increase the penetration of sulfuric acid inside the fibers, allowing to homogeneously treat the incorporated PEDOT:PSS inside each fiber and improve the electroconductivity. We found that prolonged soaking of PH1000 24H fibers in sulfuric acid at room temperature (Figure S3A) induced the appearance of new peaks (or increased their intensity) at 1154 cm^−1^ (C-O stretching), 1035 cm^−1^ (C-S stretching in thiophene ring), and 866 cm^−1^ (thiophene ring), which are associated with PEDOT:PSS. This might be associated to a higher exposure of PEDOT closer/at the surface, due to a removal of PSS [48]. However, these samples were not electroconductive. As such, we decided to heat the soaked fibers for a short time (30 min) at different temperatures (100, 130, and 150 °C) (Figure S3B) to promote a higher penetration of sulfuric acid. We found that treating the fibers with sulfuric acid at 130 °C produced the FTIR profile with the most intense peaks associated to PEDOT:PSS. We hypothesize that 130 °C is the best temperature to remove PSS, exposing more PEDOT at/closer to the surface. A more effective phase segregation between PEDOT and PSS is also likely to occur at this temperature, facilitating PSS removal [59]. We believe that this procedure favored the formation of PEDOT-enriched domains in PH1000 H_2_SO_4_ sample, which increased the electroconductivity of the fiber mats.

Treatment of PH1000 24H with heated sulfuric acid (130 °C for 30 min) (Figure 3A, spectrum (f)) induces PAN hydrolysis similarly to that observed for sample PAN H_2_SO_4_. New and unique peaks are clearly visible at 1154 cm^−1^ (C-O stretching), 1035 cm^−1^ (C-S stretching in thiophene ring), and 866 cm^−1^ (thiophene ring), which are associated with PEDOT:PSS. Peaks associated with partial PAN hydrolysis are also observed, similar to what was observed for PAN H_2_SO_4_. These include the broad band centered at 3193 cm^−1^ (O–H), at 1657 cm^−1^ (C=O), and at 1206 cm^−1^, 1096 cm^−1^, 1048 cm^−1^, and 584 cm^−1^ (CH_2_).

Raman spectroscopy (Figure 3B and Figure S4) was performed to confirm the FTIR results obtained. We could not identify relevant Raman peaks in PAN (Figure 3B, spectrum (b)). Sample PAN 24H (Figure 3B, spectrum (c)) presents new peaks at 1347 cm^−1^ (D-band) and 1552 cm^−1^ (G-band), indicating the presence of carbon-based aromatic polycyclic structures arising from thermal annealing [52,60]. A similar trend was observed for sample AI4083 and AI4083 24H (Figure S4). The relative intensity of these peaks is associated with the structural organization of cyclized PAN. For example, when comparing graphite to anthracite, graphite, which is more crystalline and electroconductive, has a high intensity G-band (≈1580 cm^−1^) and a lower intensity D-band (≈1340 cm^−1^) [61,62]. For PAN 24H, the D-band is more intense than the G-band, suggesting a higher molecular disorganization [63]. This can explain the absence of electroconductivity of thermally annealed PAN samples in the literature.

Sample PH1000 Raman spectrum (Figure 3B, spectrum (d)) presents peaks associated with the thiophene ring of PEDOT: 1086 cm^−1^, 1356 cm^−1^ and 1522 cm^−1^. In the PEDOT:PSS control sample (Figure 3B, spectrum (a)), another prominent peak is visible at 1433 cm^−1^, which is associated with the double bond found in the thiophene ring of PEDOT. After thermal annealing, sample PH1000 24H (Figure 3B, spectrum (e), and Figure S4) presents peaks at 1359 cm^−1^ (D-band) and 1546 cm^−1^ (G-band), indicating the formation of polycyclic PAN. A small peak is visible at 1450 cm^−1^, signaling the presence of PEDOT:PSS in PH1000 24H sample, as expected. Treatment with heated sulfuric acid (Figure 3B, spectrum (f)) induces further changes on the Raman spectra. New peaks are visible: 1080 cm^−1^ (HSO_4_^−^ groups), 1349 cm^−1^ (D-band), and 1570 cm^−1^ (G-band). Despite the possible influence of the PEDOT peaks for the FTIR spectrum in the same spectral region, it appears that the intensity of the G-band in PH1000 H_2_SO_4_ sample is higher than the D-band. This suggests the formation of a more ordered structure.

Overall, FTIR and Raman analyses reveal that the thermal annealing of both PAN and PAN:PEDOT:PSS fibers at 210 °C induces a transition from a linear aliphatic to a polycyclic π-conjugated structure, equivalent to that described for PAN in Figure 1 after the cyclization and the incomplete dehydrogenation steps. Moreover, the inclusion of PEDOT:PSS appears to improve the molecular organization of PAN after treatment with heated sulfuric acid, as deduced from the higher relative intensity of the G-band with respect to the intensity of the D-band.

### 3.3. Changes in Electroconductivity and Electrochemical Properties of PAN and PAN:PEDOT:PSS Fibers

The next step in our study was to investigate the consequences of the thermal annealing and sulfuric acid treatment on the electrical and electrochemical properties of PAN and PAN:PEDOT:PSS fibers. The only electroconductive samples in our study are AI4083 H_2_SO_4_ ((3.2 ± 2.2) × 10^−4^ S cm^−1^) and PH1000 H_2_SO_4_ ((3.2 ± 5.6) × 10^−3^ S cm^−1^) (Table 1). The electroconductivity values reported are slightly higher than those obtained by Babaie and colleagues [24], who reported a maximum of 1050 μS cm^−1^ (1.05 × 10^−3^ S cm^−1^) for poly(vinyl alcohol) (PVA):PEDOT:PSS 3% electrospun fibers. The values obtained are also higher than those obtained by Abedi and colleagues [64], who reported a maximum of 7.63 × 10^−3^ S m^−1^ (i.e., 7.63 × 10^−5^ S cm^−1^) for chitosan (CS):PVA:PEDOT:PSS electrospun fibers. Finally, the electroconductivity values are higher than those obtained by Park and colleagues [65] for poly(ethylene oxide) (PEO):PEDOT:PSS (1.2 × 10^−4^ S cm^−1^) and PVA:PEDOT:PSS (≈9 × 10^−6^ S cm^−1^) electrospun fibers, using Clevios^TM^ PH 1000 as the source of PEDOT:PSS. Among these studies, our PH1000 H_2_SO_4_ samples are the most electroconductive.

There are also other works in the literature that describe the development of PEDOT:PSS-based fibers with higher electroconductivities using diverse strategies. For example, the electroconductivity values attained in the current study are lower than those obtained for PEDOT:PSS-coated electrospun fibers of cellulose (472 S m^−1^, i.e., 4.72 S cm^−1^) or polybenzimidazole (28.3–147 S m^−1^, i.e., 0.283–1.47 × 10 ^−1^ S cm^−1^) fibers [66,67]. Hidayat and colleagues [41] report the production of PEDOT:PSS spin-coated PAN fibers, but no electroconductivity value is reported for comparing to this study. The main limitation on coating approaches is the difficulty to maintain the original fiber topography It should be mentioned that, electrospun PEDOT:PSS blend fibers, such as PAN:PEDOT:PSS developed in this work, overcome the detrimental effects on fiber morphology and porosity resulting from using a PEDOT:PSS coating strategy. Huang and colleagues [68] reported the production of PEO:PEDOT:PSS blend electrospun fibers with higher wet stability upon thermal annealing (130 °C for 24 h), which was proposed to induce a cross-linking of PEO with PSS. They showed that films prepared with the same blend showed an improved electroconductivity of 200 S cm^−1^ upon that annealing, though they did not report the electroconductivity of the corresponding fiber mats. Additionally, some works use wet-spinning for the production of PEDOT:PSS-based electrospun fibers. Wang and colleagues [69] reported the production of PVA:PEDOT:PSS fibers through wet spinning to a methanol coagulation bath, with electroconductivity topping 11.32 S cm^−1^ [69,70]. Liu and colleagues [39] also reported the production of PAN:PEDOT:PSS microfibers with an electroconductivity of 5 S cm^−1^, using a solution of 10% NaSCN as the coagulation bath. The authors attribute such high value to the final tempering step, responsible for PEDOT:PSS chain alignment. The electroconductivity values obtained in both wet-spinning and PEO electrospinning studies are high, but similar studies using the same PVA and PEO-based PEDOT:PSS blends for electrospinning did not yield comparatively high values [24,64,65]. Moreover, wet-spinning is a more cumbersome technique of difficult scalability, as it requires the use of a solvent coagulation bath. Finally, another strategy is the direct polymerization of chemically modified EDOT monomers (e.g., 2,5-dibromo-3,4-ethylenedioxythiophene–DBEDOT) after electrospinning. Pisuchpen and colleagues [71] describe the production of PEDOT: poly(methyl methacrylate) (PMMA) fibers with electroconductivities as high as 31.7 S cm^−1^. However, these fibers require extra synthesis steps before use, additional purification steps to remove impurities and also specialized equipment that make the process more complicated and troublesome for tissue engineering applications. Overall, it is possible to fabricate PEDOT:PSS fibers with higher electroconductivity using alternative production methods (e.g., wet spinning) and after appropriate post-processing. Comparatively to all these examples, our electrospun fibers are easily produced using commercially available reagents (PAN and PEDOT:PSS) and require simple post-processing steps to become electroconductive without affecting their morphology and/or porosity. As such, we believe our newly developed PAN:PEDOT:PSS fibers, and respective production method, to be a reliable alternative for the production of electroconductive scaffolds for (neural) tissue engineering and soft electronic applications.

We next evaluated the electrochemical responses of PAN, AI4083, and PH1000 fibers prepared under different thermal annealing conditions. We performed cyclic voltammetry using carbon SPEs, with the fibers being deposited on the working electrode. The cyclic voltammograms recorded at 0.1 V s^−1^ in 0.01 M PBS, pH 7.4, are compared in Figure 3C and Figure S5 (PAN), Figure S6 (AI4083), and Figure S7 (PH1000).

The increase in thermal annealing time of PAN fibers (Figure S5) leads to a maximum electrochemical activity for PAN 24H, assessed by the cyclic voltammogram profile. This is attributed to the formation of a polycyclic structure in PAN 24 H, which favors electronic conductivity [72,73,74]. The electrochemical activity remains very similar for the other thermal annealing times. For AI4083 fibers (Figure S6), there are no noticeable changes in electrochemical activity with thermal annealing time, with all voltammograms exhibiting a very similar shape and size. The incorporation of PEDOT:PSS Clevios^TM^ P VP AI 4083 in PAN did not affect the electrochemical properties of the obtained PAN:PEDOT:PSS fibers.

The electrochemical activity of PH1000 fibers (Figure 3C) appears to decrease with increasing thermal annealing time. Sample PH1000 24H fibers show some recovery of electrochemical activity, as evidenced by the increase in voltammogram peak intensity and area (Figure 3C), when compared with PH1000. The voltammogram area of PH1000 24H is higher when compared with the one of the samples PAN and PAN 24H, indicating an improvement in electrochemical activity with the incorporation of PEDOT:PSS Clevios^TM^ PH 1000 in PAN. Finally, electrochemical activity is also improved when compared to samples PH1000 1H, PH1000 5H, and PH1000 10H (Figure S7). Sample PH1000 H_2_SO_4_ evidences a great improvement in electrochemical activity associated with heated sulfuric acid treatment of sample PH1000 24H. The removal of PSS^-^ has been associated to the increase in electroconductivity of PEDOT after treatment with sulfuric acid. It is possible that replacement of PSS^-^ by HSO_4_^−^/SO_4_^2−^, as the doping agent [8,75], may lead to a conformational change in PEDOT from coil to a linear conformation [63]. Additionally, a small anion is less likely to impair the PEDOT electronic conductivity when compared with the long chains of the polyanion (PSS^-^). At variance with PSS^-^ chains, the sulfate ions are mobile and may improve the ionic conductivity of the fibers, complementing the increase of the PEDOT electronic conductivity [76].

### 3.4. Influence of Annealing and Sulfuric Acid Treatment on Spin-Coated PEDOT:PSS Films

**Figure 4:**
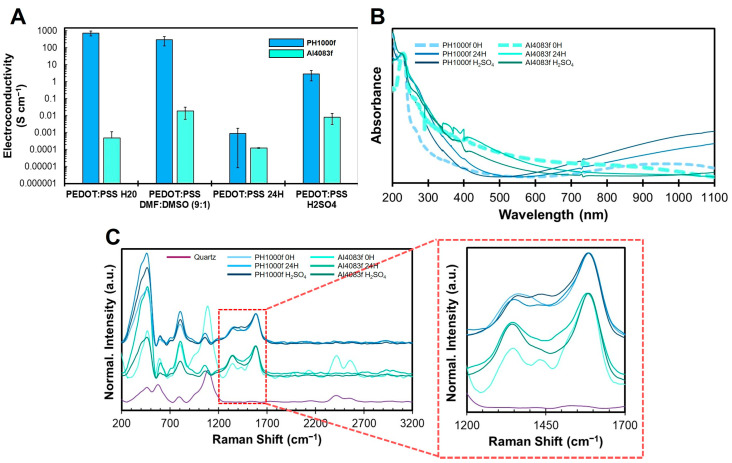
Physico–chemical assessment of the stability of PEDOT:PSS spin-coated films, obtained under similar conditions used for PAN:PEDOT:PSS fibers processing: (**A**) Electroconductivity (mean ± std, *n* = 3) of spin-coated films of PEDOT:PSS Clevios^TM^ PH 1000 (PH1000f) and Clevios^TM^ P VP AI 4083 (AI4083f) after different processing methods. (**B**) UV/Vis (normalized to the maximum peak in the 225–235 nm region) and (**C**) Raman spectra (normalized to the maximum peak found in the 1200–1700 cm^−1^ region) of spin-coated PEDOT:PSS films obtained from PEDOT:PSS dispersion in DMF:DMSO (9:1) (PH1000f 0H and AI4083f 0H), thermal annealing (PH1000f 24H and AI4083f 24H), and treatment with heated sulfuric acid (PH1000f H_2_SO_4_ and AI4083f H_2_SO_4_).

PAN:PEDOT:PSS fibers can be considered a complex system. The predominance of PAN in the fibers does not allow to directly assess the changes that occur in the PEDOT:PSS sub-system. As such, we used spin-coated PEDOT:PSS films, prepared from a dispersion in DMF-DMSO containing 10% DMSO, to study the effect of processing conditions directly on PEDOT:PSS. The results obtained are summarized in Figure 4. The electroconductivity (Figure 4A) of spin-coated PEDOT:PSS Clevios^TM^ PH 1000 films (730 ± 280 S cm^−1^—sample PH1000f) from the as received aqueous dispersion is higher than the corresponding sample produced using PEDOT:PSS Clevios^TM^ P VP AI 4083 ((4.9 ± 6.3) × 10^−4^ S cm^−1^—Sample AI4083f). These results match observations from other works [6,9].

The electroconductivity of films produced after redispersion of PEDOT:PSS in DMF:DMSO (9:1) changed slightly with respect to that of the films prepared with the as-received aqueous dispersions, with sample PH1000f decreasing to 297 ± 167 S cm^−1^ and sample AI4083f increasing to (1.7 ± 1.0) × 10^−2^ S cm^−1^. Both DMF and DMSO are solvents described as having positive effects on the electroconductivity of PEDOT:PSS, including enhancement of electroconductivity through pseudo-doping effect and promoting PEDOT segregation from PSS [9]. As such, redispersion of PEDOT:PSS in the DMF:DMSO solvent mixture could possibly affect the properties of PEDOT:PSS. Our results indeed show that redispersion of PEDOT:PSS in DMF:DMSO (9:1) does not significantly affect the conductivity of PH1000, but it improves significantly the electroconductivity of AI4083 films. The higher PSS content of AI4083 PEDOT:PSS formulation is likely to originate a more pronounced effect of the insulating PSS segregation and, consequently, on the film electroconductivity increase upon redispersion in DMF-DMSO.

Heat treatment at 210 °C for 24 h negatively impacted the electroconductivity of both sample PH1000f ((9.0 ± 8.9) × 10^−4^ S cm^−1^) and sample AI4083f ((1.2 ± 0.1) × 10^−4^ S cm^−1^) (identified in Figure 4A as PEDOT:PSS 24H). Several studies using thermogravimetric analysis show that PEDOT:PSS is thermically stable at 210 °C [77,78]. Moreover, thermal treatment at 150–200 °C (maximum of 20 min) is routinely used for the drying and thermal annealing of PEDOT:PSS films to improve both electroconductivity and crystallinity [79,80]. However, prolonged thermal annealing can be detrimental to the electroconductivity of PEDOT:PSS. Bontapalle and colleagues [81] reported that thermal annealing at 150 °C of PEDOT:PSS spin-coated films, prepared from commercially available formulations, induces a decrease in electroconductivity by 1000 × within ≈24 h, while this rate is proportionally slower with lower temperatures (e.g., ≈68 h for 120 °C). When thermal annealing was conducted at 200 °C for 24 h, the authors observed a decrease in absorption in the 900–1250 nm region and an increase at 600 nm. The authors correlate these observations with a change in PEDOT charge (reduction), from bipolaron to a neutral stage. This effect is usually associated with the presence of atmospheric oxygen [78,81,82], although in some studies these changes can also happen in an inert atmosphere [80]. When the same authors performed a similar study at 120 °C (≈24 h), they observed by AFM an increase in PSS segregation from PEDOT and the shrinkage of PEDOT area in the film, reducing electroconductivity.

Treatment with sulfuric acid allowed to partly recover the electroconductivity of both samples PH1000f (2.8 ± 1.7 S cm^−1^) and AI4083f ((8.2 ± 5.1) × 10^−3^ S cm^−1^). Statistical analysis was conducted to evaluate differences between the electroconductivities of the different PEDOT:PSS spin-coated films obtained. Although ANOVA test suggested the existence of statistically significant differences between samples (*p*-value = 6 × 10^−7^, F = 21.1 > F_critical_ = 2.7), post hoc analysis did not evidence strong differences between the samples. Nevertheless, changes in electroconductivity of the different PEDOT:PSS films were accompanied by structural changes in the PEDOT:PSS system. We will explore more in depth such changes in the next paragraphs.

Considering the observations of Bontapalle and colleagues [81], we hypothesize that the electroconductivity rescue observed upon sulfuric acid treatment involves a change in PEDOT charge (oxidation state), with an increase of bipolaronic states density, relaxation of PEDOT chains and the removal of PSS from the film. These observations are supported by our UV/Vis results (Figure 4B). Thermal annealing decreases the absorption in the NIR region (1000–1100 nm) does not greatly affect the absorption profile of AI4083f, while increasing absorption in the NIR region (1000–1100 nm) of sample PH1000f. When these samples are treated with sulfuric acid, absorption in the region 700–1100 nm for sample PH1000 greatly increases, though that of AI4083 shows only a small increase—and in the region 1000–1100 nm. These results suggest the treatment with sulfuric acids increases the absorption of the conductive states in PEDOT, especially in sample PH1000f, which also leads to PEDOT chain relaxation and partly explains the improvement observed in electroconductivity. It is also possible that the increase of the bipolaronic spatial extension, resulting from a relaxation of the PEDOT chains, may also increase the light absorption.

Raman analysis also reveal changes on PEDOT:PSS after treatment with sulfuric acid. Sample PH1000f 0H (Figure 4C) presents 2 main Raman peaks at 1360 cm^−1^ (attributed to C_β_–C_β_) and 1594 cm^−1^ (attributed to C_α_=C_β_ asymmetric stretching and C=C of PSS), similar to what was observed by Lin and colleagues [83]. After thermal annealing for 24 h at 210 °C, the peak at 1360 cm^−1^ red-shifts to 1353 cm^−1^ and decreases in intensity. The weakening of the C_β_–C_β_ bond, is consistent with an increased predominance of a benzenoid-like structure of the thiophene ring, which could be associated to a reduction of the PEDOT oxidation state. Additionally, a small peak at appears at 1426 cm^−1^ (C_α_=C_β_ symmetric stretching), which seems to be the result of a blue-shift of a shoulder seen at about 1400 cm^−1^ in the initial film. After treatment with heated sulfuric acid, the peak at 1353 cm^−1^ blue-shifts back to 1360 cm^−1^ (attributed to C_β_–C_β_) and increases in intensity, the peak at 1426 cm^−1^ (C_α_=C_β_ symmetric stretching) blue-shifts to 1440 cm^−1^ and significantly increases in intensity. The main peak at 1594 cm^−1^ (attributed to C_α_=C_β_ asymmetric stretching and C=C of PSS) remains at the same energy. In conclusion, the treatment with heated sulfuric acid, leaves unchanged the energy of the main absorption peak and appears to decrease the energy of the C_β_–C_β_ bond. This is somewhat surprising as an increase of the C_β_–C_β_ bond energy should be accompanied by a decrease of the C_α_–C_β_ bond energy. These results do not provide a clear confirmation of a change in the oxidation state of the PEDOT chains upon thermal and/or sulfuric acid treatment [81,84]. The variations in the electroconductivity of the spin coated PEDOT:PSS films are therefore more likely related to changes of the samples microstructures in view of the phase separation between PEDOT and PSS domains and to removal of the electronically insulating PSS which limits the contacts between the conducting PEDOT grains/crystals. We hypothesize that after sulfuric acid treatment, PEDOT also adopts a more linear conformation, leading to higher delocalization of the quinoid structures, which favors electroconductivity [49]. This is also supported by our UV/Vis results (Figure 4B).

The Raman spectra of sample AI4083f presents 3 peaks at frequencies of 1349 cm^−1^ (C_β_–C_β_), 1431 cm^−1^ (C_α_=C_β_ symmetric stretching), and 1590 cm^−1^ (C_α_=C_β_ asymmetric stretching and C=C of PSS). Thermal annealing induces an increase in intensity of the peak at 1349 cm^−1^ and the disappearance of the peak at 1431 cm^−1^. This profile slightly changes after treatment with sulfuric acid, and the peak at 1431 cm^−1^ is not visible anymore. It is possible the spectral changes observed in sample PH1000 are not so clearly visible in sample AI4083, due to the high amount of PSS (peak at ≈1590–1600 cm^−1^), which may mask the PEDOT spectrum.

Overall, our results with spin-coated PEDOT:PSS films show that the combined thermal annealing and heated sulfuric acid treatment affect the electroconductivity and structure of PEDOT:PSS. Sample PH1000 evidenced the most dramatic changes on electroconductivity and structure of PEDOT. The treatment with heated sulfuric acid enabled the partial rescue of sample PH1000f. As such, we deduce that the increase in electroconductivity observed for PAN:PEDOT:PSS fibers PH1000 H_2_SO_4_ was due to a direct positive effect on PEDOT:PSS.

### 3.5. Mechanical Properties of PAN and PAN:PEDOT:PSS Fibers

**Figure 5:**
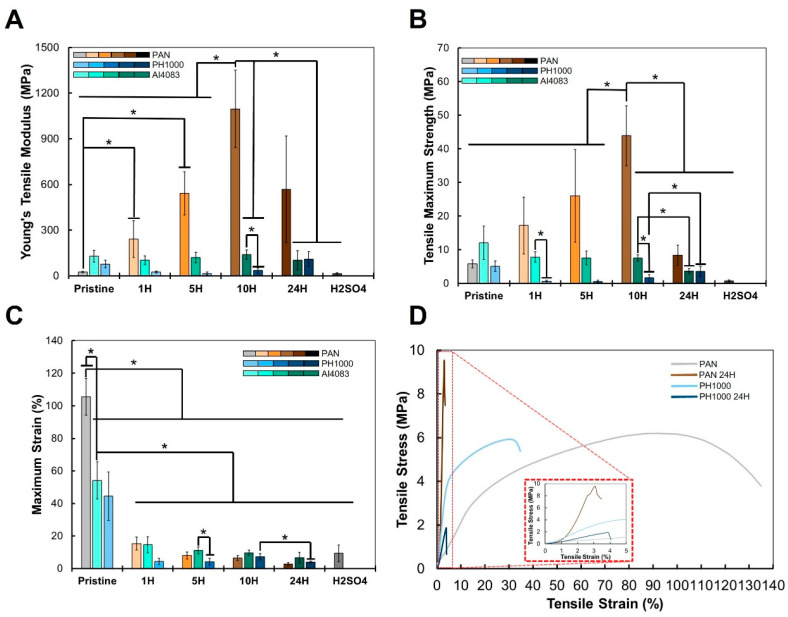
Mechanical properties of the PAN and PAN/PEDOT:PSS fibers. (**A**) Young’s tensile modulus, (**B**) maximum strain, and (**C**) tensile maximum strength (mean ± std, *n* = 5, (*) means *p* < 0.05). (**D**) Representative stress–strain curves obtained for the main samples of this study.

The next step in our study was to determine the mechanical properties of the obtained PAN and PAN:PEDOT:PSS fibers and evaluate how they change with thermal annealing and treatment with heated sulfuric acid. The main results are summarized in Figure 5 and Figure S8.

The most drastic changes in mechanical properties are observed for PAN fibers upon thermal annealing. We expected the formation of a more rigid polycyclic structure, which would make the fibers stiffer, yet brittle and fragile. Indeed, the Young’s modulus (Figure 5A) initially increased with time of thermal annealing, from PAN (25 ± 5 MPa) to PAN 1H (241 ± 122 MPa), PAN 5H (541 ± 142 MPa), and PAN 10H (1096 ± 255 MPa). However, the Young’s modulus then decreases to 568 ± 350 MPa for the PAN 24H sample, and further decreases to 15 ± 6 MPa for the PAN H_2_SO_4_ sample. Such a trend was not followed by thermally annealed AI4083 and PH1000 samples. Overall, there was no statistically significant change on the Young’s modulus of samples AI4083 (129 ± 39 MPa) and AI4083 24H (103 ± 63 MPa). However, these values were lower than those observed for the respective PAN fibers. For PH1000 samples, from the original PH1000 fibers (78 ± 25 MPa) there was a small decrease for samples PH1000 1H (25 ± 6 MPa) and PH1000 5H (15 ± 9 MPa), only to increase for samples PH1000 10H (36 ± 18 MPa) and PH1000 24H (110 ± 49 MPa). The obtained Young’s tensile modulus for the fibers is significantly higher than that of normal neural tissue (0.4–2 kPa) [85,86]. The obtained values match those of meninges, a membrane that separates neural tissue from bone tissue and assists in providing support in complex tissues. For example, bovine pia-mater, which directly contacts neural tissue, is described to have tensile strength of 14–26 MPa [87]. Other studies provide similar insights to the mechanical properties of surrounding tissues in direct contact with neural cells [88,89,90]. As such, the mechanical properties of the obtained fibers are not a limitation to their application in neural TE.

Other changes in the mechanical properties of PAN and PAN:PEDOT:PSS fibers were observed. The tensile maximum strength (Figure 5B) of PAN fibers (5.8 ± 1.2 MPa) also increased with increasing thermal annealing time. The highest value was observed for sample PAN 10H (43.9 ± 8.9 MPa), and then decreased for sample PAN 24H (8.4 ± 3.0 MPa) and PAN H_2_SO_4_ (0.8 ± 0.3 MPa). These changes accompanied those of Young’s modulus but were not overall expected. We currently do not have an explanation to this phenomenon.

Changes in tensile maximum strength of PAN:PEDOT:PSS fibers are variable. There is a gradual decrease from sample AI4083 (12.1 ± 5.0 MPa) to sample AI4083 24H (3.7 ± 0.7 MPa). A similar gradual decrease is also observed from sample PH1000 (5.1 ± 1.5 MPa) to sample PH1000 24H (3.5 ± 1.6 MPa).

There is an overall decrease of the obtained maximum strain (Figure 5C) for both PAN and PAN:PEDOT:PSS fibers. Thermal annealing of PAN (106 ± 11%) greatly reduces the maximum strain, bottoming with PAN 24H (3 ± 1%), and then increasing slightly for PAN H_2_SO_4_ (9 ± 5%). Incorporation of PEDOT:PSS into PAN reduces the maximum strain for samples of AI4083 (54 ± 11%) and PH1000 (44 ± 15%). After thermal annealing, maximum strain further decreases, bottoming in samples AI4083 24H (7 ± 3%) and PH1000 24H (4 ± 1%).

Overall, the mechanical properties of the obtained fibers were greatly affected by the incorporation of PEDOT:PSS and processing steps taken. Thermal annealing of PAN fibers increased their stiffness up to 10 h treatment. Incorporation of PEDOT:PSS in PAN made the obtained samples softer yet more fragile. Similar observations were made by Li and colleagues [40] for wet-spinned PAN:PEDOT:PSS fibers. It was not possible to evaluate the mechanical properties of samples AI4083 H_2_SO_4_ and PH1000 H_2_SO_4_ due to quick breaking when mounted at the machine. However, the samples were easily manipulated, and we were able to conduct further assays.

### 3.6. Biocompatibility of PAN and PAN:PEDOT:PSS Fibers

**Figure 6:**
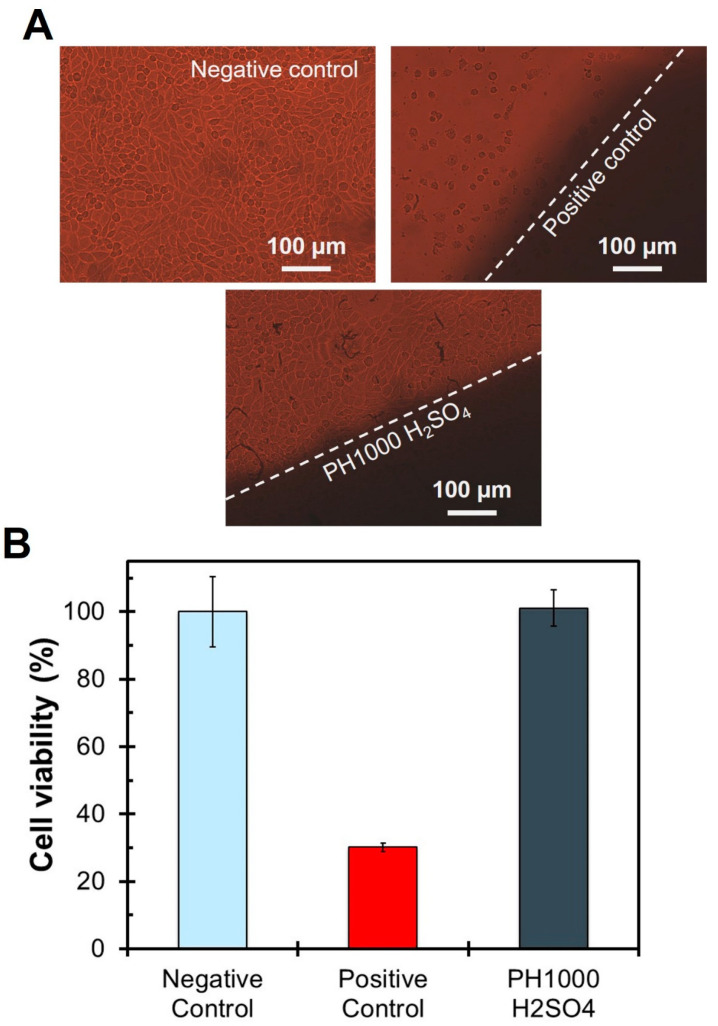
Summary of fiber biocompatibility evaluation (L-929 mouse fibroblasts) following the ISO10993 guidelines for PH1000 H_2_SO_4_ fibers. (**A**) microscopy images depicting the direct contact test results (48 h) evidencing the negative control (regular culture medium), the positive control (latex) and sample PH1000 H_2_SO_4_. (**B**) MTT assay results expressed as percentage of viable cells after 48 h of cell incubation with the lixiviates (mean ± std, *n* = 3).

The existing measurements of biocompatibility in the literature for PAN-based biomaterials were either made of pristine PAN [38] or graphitized PAN [37,91]. As such, it was important to investigate the biocompatibility of our new PAN:PEDOT:PSS materials to evaluate their potential biomedical applications. The evaluation of the biocompatibility of the obtained PAN and PAN:PEDOT:PSS fibers was performed following the ISO-10993 guidelines. The obtained results are summarized in Figure 6 and Figure S9. We focused our analysis on the PAN:PEDOT:PSS composites, in particular sample PH1000 H_2_SO_4_, the most electroconductive in the study, as no work in the literature had done so.

Our results indicate that PAN fibers are biocompatible regarding their lixiviates (77 ± 10%) (Figure S9A), and no visible halo of inhibition was observed around the material when there is direct contact of the fibers with cultured fibroblasts (Figure S9B). Incorporation of PEDOT:PSS in the fibers did not affect their biocompatibility, with samples AI4083 (112 ± 1%) and PH1000 (117 ± 8%) presenting high cell viability values above the cytotoxicity limit of 70% established by ISO-10993 guidelines (Figure S9C). Moreover, no visible halo of inhibition was observed for these samples (Figure S9D). The thermal annealing of samples AI4083 and PH1000 lead to a slight decrease in cell viability, bottoming in samples AI4083 24H (85 ± 6%) and PH1000 24H (83 ± 3%), with no inhibition halo being observed. Overall, we can consider that incorporation of PEDOT:PSS in PAN improves its biocompatibility, even after thermal annealing.

Finally, we evaluated the biocompatibility of sample PH1000 H_2_SO_4_ (Figure 6), the most electroconductive sample in our study. The obtained viability was 101 ± 5%, higher than the cytotoxicity limit of 70% required in ISO standards. Moreover, no evidence of cell death, inhibition halo ad/or changes on cell morphology were observed. These results suggest that sample PH1000 H_2_SO_4_ is biocompatible and that treatment of PAN:PEDOT:PSS fibers with sulfuric acid has no evident negative consequences on biocompatibility.

### 3.7. Proliferation Assay with NSCs

**Figure 7:**
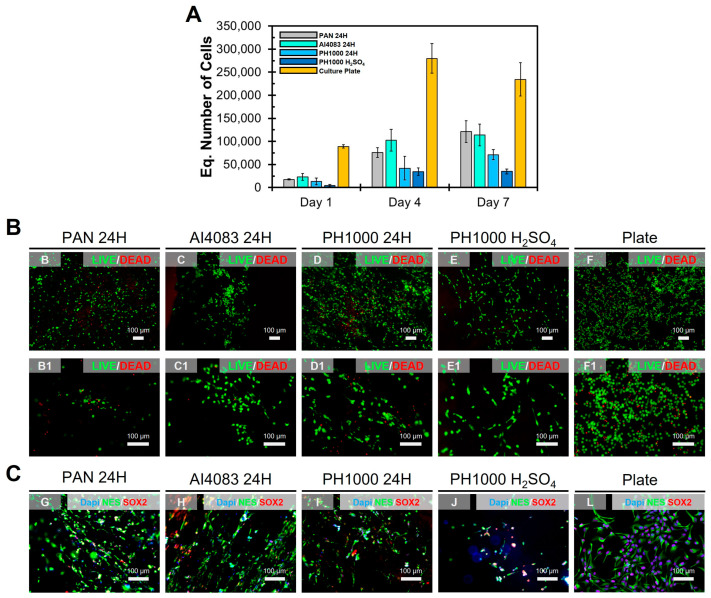
Proliferation assay (ReNCell-VM cells) results for the main fibers obtained in this study. (**A**) Cell growth curve (mean ± std, *n* = 3). (**B**) LIVE/DEAD staining at day 7. (**C**) immunostaining of SOX2 (red—stem cell marker), NES (green—neural stem cell marker) and DAPI counter-stain (blue—Nucleus) at day 7.

The biocompatibility results obtained suggest that PAN:PEDOT:PSS fibers can be suitable for biomedical applications. The final step in our work was to test the suitability of PAN:PEDOT:PSS fibers for neural TE applications. For this, we performed a proliferation assay using an immortalized human cell line of neural stem cells (NSCs): ReNCell-VM. The biocompatibility and suitability for TE applications were evaluated using a proliferation assay, similar to what was performed for other biomaterials [20,46]. The main results are summarized in Figure 7 and Figure S11 and Table 2.

NSCs were seeded on samples PAN 24H, AI4083 24H, PH1000 24H, and PH1000 H_2_SO_4_, and some cells were seeded on culture plate as controls. Alamar^TM^ Blue assay was used to monitor cell metabolic activity on the samples (Figure 7A), which was used to calculate the equivalent cell number present through a calibration curve (Figure S10). From here, we calculated cell kinetic parameters summarized on Table 2. We found that cell adhesion was very similar between samples PAN 24H (27 ± 2%), AI4083 24H (34 ± 11%), and PH1000 (20 ± 11%). Sample PH1000 H_2_SO_4_ had the lowest adhesion value of the test (7 ± 4%), suggesting that treatment of PAN:PEDOT:PSS fibers with heated sulfuric acid had a negative impact on its surface properties. However, sample PH1000 H_2_SO_4_ had the highest growth rate (0.37 ± 0.11 day^−1^) and the lowest doubling time (49 ± 14 h) of the study. We observed a similar trend in our previous work [20] with poly(caprolactone):poly(aniline):camphorsulfonic acid (PCL:PANI:CSA) electrospun fibers. Although cell adhesion was compromised by higher amounts of PANI:CSA, cell proliferation resumed throughout the duration of the assay. These results indicate that normal cell growth is not compromised in sample PH1000 H_2_SO_4_.

Finally, we studied the morphology of NSCs at day 7. All cells showed a normal spindle-like morphology, confirming that the support material was biocompatible. Moreover, LIVE/DEAD staining (Figure 7B) shows that most of cultured cells in contact with the tested fibers are viable. Finally, immunofluorescence analysis reveals that NSCs express normal pluripotency markers (Figure 7C and Figure S11), including SRY-Box Transcription Factor 2 (SOX2—stem cells) and Nestin (NES–NSCs). Overall, our results show that NSCs growing on samples PAN 24H, AI4083 24H, PH1000 24H, and PH1000 H_2_SO_4_ present a normal morphology at day 7. These results confirm our previous observations regarding biocompatibility. They also evidence an intimate interaction between cells and the supporting material without significant changes to the normal NSCs phenotype.

## 4. Conclusions

In this study, we produced electrospun PAN:PEDOT:PSS blend fibers using a unique strategy not yet approached by other research groups. PEDOT:PSS incorporation in PAN was possible after its redispersion in DMF:DMSO (9:1), and electrospun fibers were successfully produced. Thermal annealing (210 °C) and treatment with heated sulfuric acid (30 min) allowed to obtain electroconductive PAN:PEDOT:PSS blend fibers. The processing conditions influenced the structure and electrochemical properties of both PAN and PEDOT:PSS. Sample PH1000 H_2_SO_4_, which had the highest electroconductivity in our study (3.2 × 10^−3^ S cm^−1^), is biocompatible and allowed the proliferation of NSCs at its surface. Future work should focus on fiber optimization, including further electrospinning optimization, complemented by rheological analysis of obtained solutions, and influence of production and processing parameters on fiber morphology, including fiber diameter and porosity, physico–chemical properties, and electroconductivity.

For the first time, we describe the development of electroconductive PAN:PEDOT:PSS electrospun blend fibers. These fibers are among the most electroconductive reported in the literature; moreover, they are electrochemically active and suitable for neural applications. Overall, the produced blend PAN:PEDOT:PSS fibers hold great promise for neural TE and other biomedical applications, including electrodes for electrical stimulation and devices for soft electronics.

## Figures and Tables

**Table 1:** Summary of the sample code and respective properties of the main fibers obtained in this study, including average fiber diameter (*n* = 50–100) and electroconductivity (*n* = 5). ^(^*^)^ means *p* < 0.05 when compared with PAN. ^(a)^ means *p* < 0.05 when compared with PAN 24H. ^(b)^ means *p* < 0.05 when compared with PAN H_2_SO_4_. ^(c)^ means *p* < 0.05 when compared with AI4083. ^(d)^ means *p* < 0.05 when compared with AI4083 24H. ^(e)^ means *p* < 0.05 when compared with AI4083 H_2_SO_4_. ^(f)^ means *p* < 0.05 when compared with PH1000. ^(g)^ means *p* < 0.05 when compared with PH1000 24H.

Sample Description	Sample Code	Source of PEDOT:PSS Used	Heat Treatment (210 °C)	Sulfuric Acid Treatment	Fiber Diameter (nm)	Electroconductivity (S cm ^−1^)
PAN pristine	PAN	(not used)	no	no	640 ± 159	(not electroconductive)
PAN 24 h	PAN 24H	(not used)	yes	no	1100 ± 135 ^(^*^)^	(not electroconductive)
PAN 24 h + H_2_SO_4_	PAN H_2_SO_4_	(not used)	yes	yes	612 ± 107 ^(a)^	(not electroconductive)
PAN:PEDOT:PSS AI4083 pristine	AI4083	CleviosTM PVP AI 4083	no	no	356 ± 104 ^(^*^)(a)(b)^	(not electroconductive)
PAN:PEDOT:PSS AI4083 24 h	AI4083 24H	CleviosTM PVP AI 4083	yes	no	592 ± 117 ^(^*^)(a)(c)^	(not electroconductive)
PAN:PEDOT:PSS AI4083 24 h + H_2_SO_4_	AI4083 H_2_SO_4_	CleviosTM PVP AI 4083	yes	yes	507 ± 125 ^(^*^)(a)(b)(c)(d)^	(3.2 ± 2.2) × 10^−4^
PAN:PEDOT:PSS PH1000 pristine	PH1000	CleviosTM PH 1000	no	no	515 ± 120 ^(^*^)(a)(b)(c)^	(not electroconductive)
PAN:PEDOT:PSS PH1000 24 h	PH1000 24H	CleviosTM PH 1000	yes	no	437 ± 109 ^(^*^)(a)(b)(c)(d)(e)(f)^	(not electroconductive)
PAN:PEDOT:PSS PH1000 24 h + H_2_SO_4_	PH1000 H_2_SO_4_	CleviosTM PH 1000	yes	yes	940 ± 210 ^(^*^)(a)(b)(c)(d)(e)(f)(g)^	(3.2 ± 5.6) × 10^−3^

**Table 2:** Summary of the cell dynamics parameters obtained in the proliferation assay.

Sample	Adhesion (%)	Growth Rate (Day^−1^)	Doubling Time (h)
PAN 24H	27 ± 2	0.32 ± 0.03	53 ± 5
AI4083 24H	34 ± 11	0.27 ± 0.05	62 ± 10
PH1000 24H	20 ± 11	0.30 ± 0.07	59 ± 14
PH1000 H_2_SO_4_	7 ± 4	0.37 ± 0.11	49 ± 14
Culture Plate	134 ± 6	0.16 ± 0.02	106 ± 13

## Data Availability

Data is available on request.

## Supplementary Materials

The following supporting information can be downloaded at: https://www.mdpi.com/article/10.3390/polym15132760/s1. Figure S1: SEM images and respective size distribution histograms for all the fibers obtained in this study; Figure S2: FTIR spectra of samples; Figure S3: FTIR spectra of different PH1000 H2SO4 samples at different stages of optimization; Figure S4: Raman spectra for the main electrospun samples obtained in this study; Figure S5: Cyclic voltammetry profiles of samples produced using pristine PAN; Figure S6: Cyclic voltammetry profiles of samples produced using PAN blended with PEDOT:PSS CleviosTM P VP AI 4083; Figure S7: Cyclic voltammetry profiles of samples produced using PAN blended with PEDOT:PSS CleviosTM PH 1000; Figure S8: Examples of stress-strain curves for all samples; Figure S9: Summary of fiber biocompatibility evaluation following the ISO10993 guidelines for pristine PAN fibers; Figure S10: Alamar Blue® calibration curve for ReNCell-VM and used to calculate the equivalent number of cells in the proliferation assay; Figure S11: Immunostaining (split channels and respective merge) of ReNCell-VM after proliferat-ing for 7 days on different samples: SOX2 (red—stem cell marker), NES (green—neural stem cell marker) and Dapi counter-stain (blue—Nucleus); Table S1: Summary of the sample code and respective average fiber diameter (*n* = 50–100) of all the samples obtained in this study.
