# Supplementary material for: Production of Blended Poly(acrylonitrile): Poly(ethylenedioxythiophene):Poly(styrene sulfonate) Electrospun Fibers for Neural Applications
Source: Polymers (Basel). 2023 Jun 21;15(13):2760. doi: 10.3390/polym15132760 (PMC10346782; doi:10.3390/polym15132760)
Supplement: Supplementary file 1 [file polymers-15-02760-s001.zip › polymers-2417659-supplementary.pdf]

Supplementary Information

Production of Blended Poly(acrylonitrile): Poly(ethylenedioxythiophene):Poly(styrene sulfonate) Electrospun Fibers for Neural Applications

Fábio F. F. Garrudo [1,2,3,4,*], Giulia Filippone [2,3], Leonor Resina [2,3,4,5], João C. Silva [2,3,4], Frederico Barbosa [2,3,4], Luís F. V. Ferreira [3,4,6], Teresa Esteves [3,4], Ana Clara Marques [6,7], Jorge Morgado [1,2,*] and Frederico Castelo Ferreira [2,3,4,*]

1 Instituto de Telecomunicações, Instituto Superior Técnico, Universidade de Lisboa, Avenida Rovisco Pais, 1049-001 Lisboa, Portugal
2 Department of Bioengineering, Instituto Superior Técnico, Universidade de Lisboa, Avenida Rovisco Pais, 1049-001 Lisboa, Portugal; joao.f.da.silva@tecnico.ulisboa.pt (J.C.S.)
3 iBB—Institute for Bioengineering and Biosciences, Instituto Superior Técnico, Universidade de Lisboa, Avenida Rovisco Pais, 1049-001 Lisboa, Portugal
4 Associate Laboratory i4HB—Institute for Health and Bioeconomy, Avenida Rovisco Pais, 1049-001 Lisboa, Portugal
5 Departament d'Enginyeria Química and Barcelona Research Center for Multiscale Science and Engineering, EEBE, Universitat Politècnica de Catalunya, 08019 Barcelona, Spain
6 Department of Chemical Engineering, Instituto Superior Técnico, Universidade de Lisboa, Avenida Rovisco Pais, 1049-001 Lisboa, Portugal
7 CERENA, DEQ, Instituto Superior Técnico, Universidade de Lisboa, Avenida Rovisco Pais, 1049-001 Lisboa, Portugal
* Correspondence: fabio.garrudo@tecnico.ulisboa.pt (F.F.F.G.); jmfmorgado@tecnico.ulisboa.pt (J.M.); frederico.ferreira@tecnico.ulisboa.pt (F.C.F.)

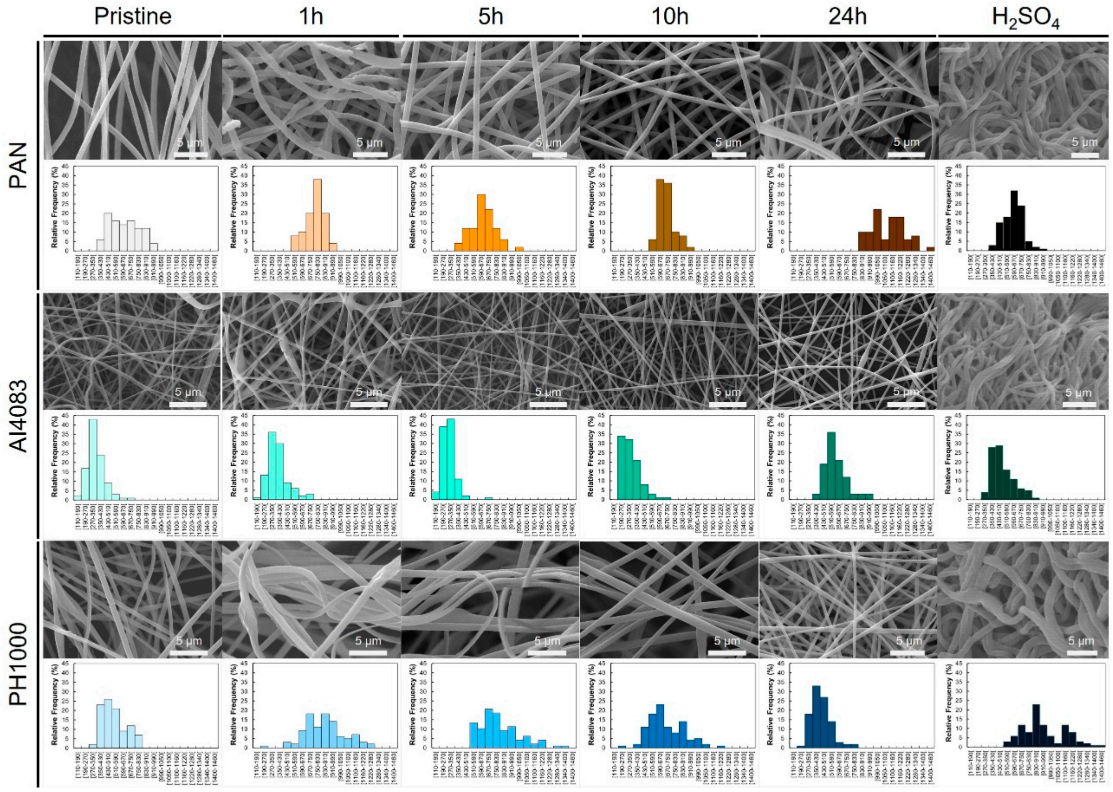

Figure S1. SEM images and respective size distribution histograms for all the fibers obtained in this study.

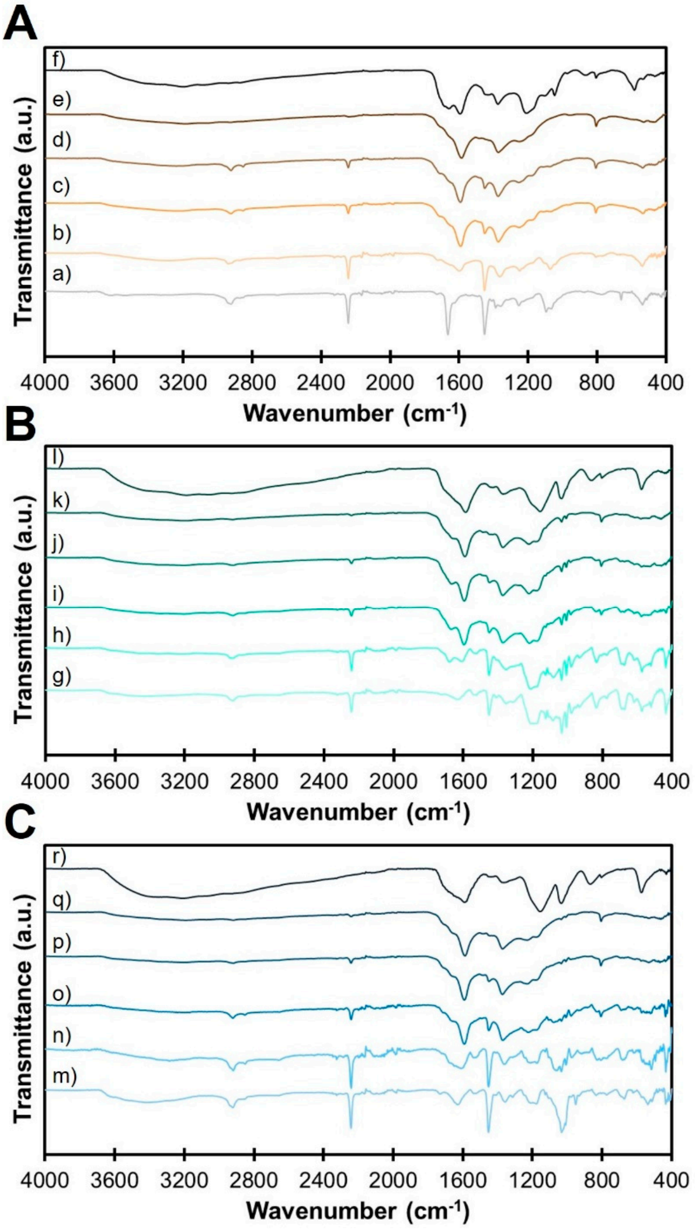

Figure S2. FTIR spectra of samples produced using (**A**) pristine PAN or PAN blended with (**B**) PEDOT:PSS AI4083 or (**C**) PEDOT:PSS PH1000. Sample code is **a**) PAN, **b**) PAN 1H, **c**) PAN 5H, **d**) PAN 10H, **e**) PAN 24H, **f**) PAN H_2SO_4, **g**) AI4083, **h**) AI4083 1H, **i**) AI4083 5H, **j**) AI4083 10H, **k**) AI4083 24H, **l**) AI4083 H_2SO_4, **m**) PH1000, **n**) PH1000 1H, **o**) PH1000 5H, **p**) PH1000 10H, **q**) PH1000 24H, and **r**) PH1000 H_2SO_4.

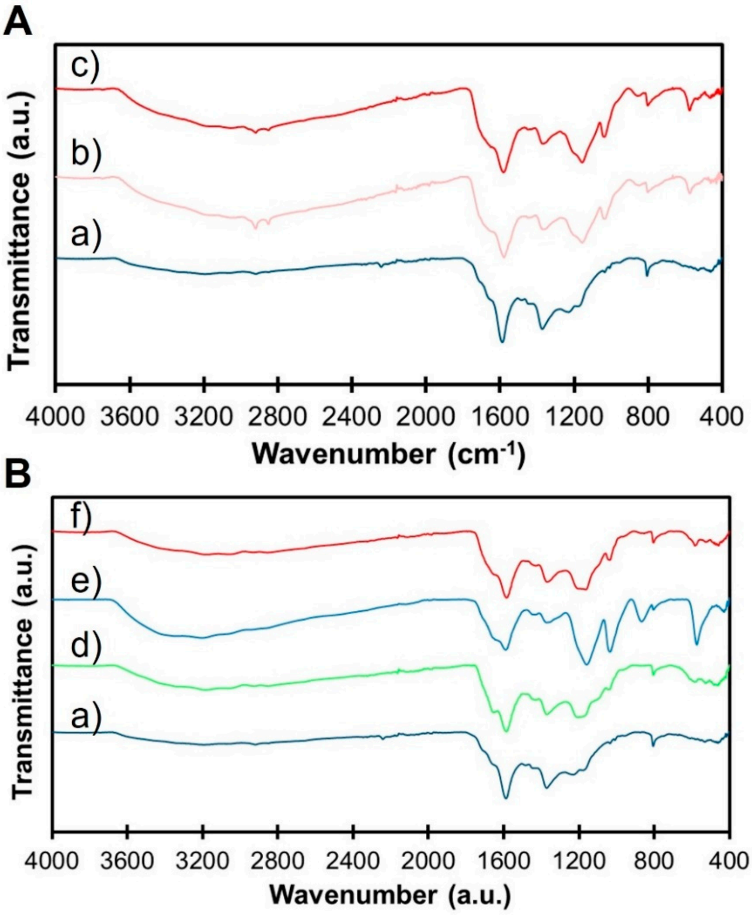

Figure S3. FTIR spectra of different PH1000 H_2SO_4 samples at different stages of optimization. (**A**) Different incubation times in sulfuric acid at RT, **a**) zero hours, **b**) 1 hour, **c**) 24 hours. (**B**) Different temperature of the 30 min long incubation in sulfuric acid, **d**) 100ºC, **e**) 130 ºC, **f**) 150 ºC.

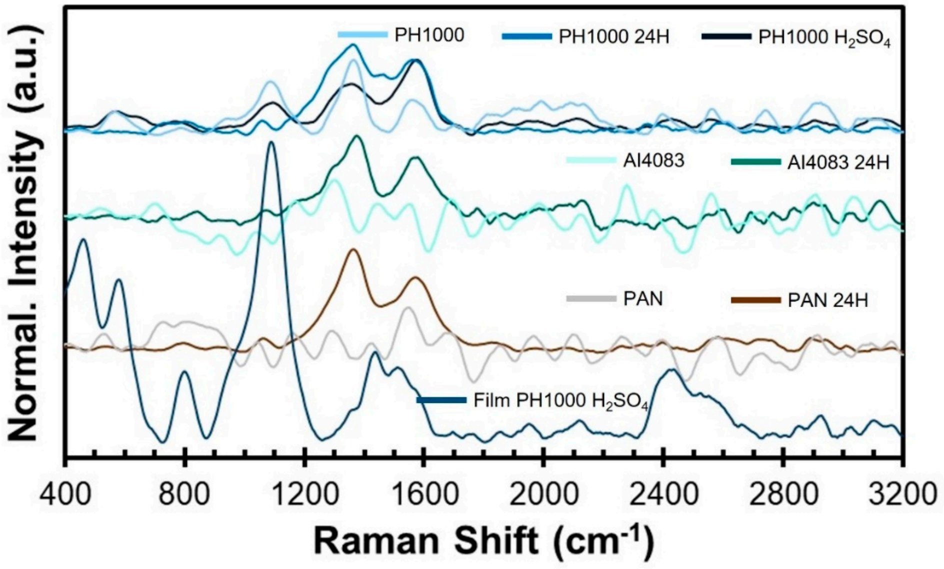

Figure S4. Raman spectra for the main electrospun samples obtained in this study.

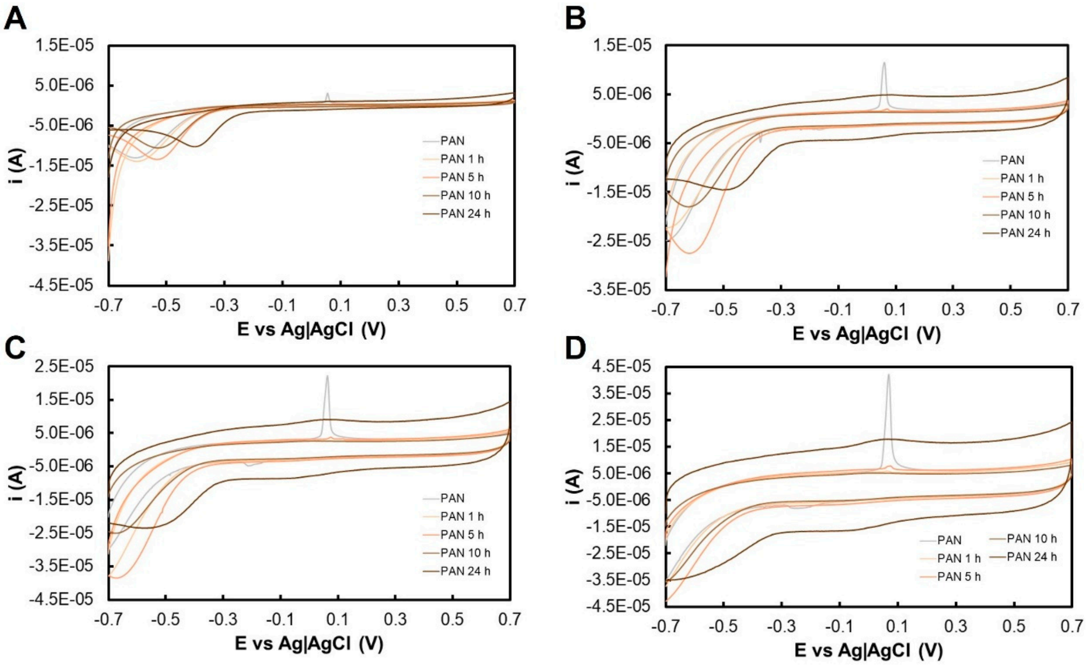

Figure S5. Cyclic voltammetry profiles of samples produced using pristine PAN. Different scanning speeds were tested: (**A**) 0.01 Vs, (**B**) 0.05 Vs, (**C**) 0.1 Vs, (**D**) 0.2 Vs.

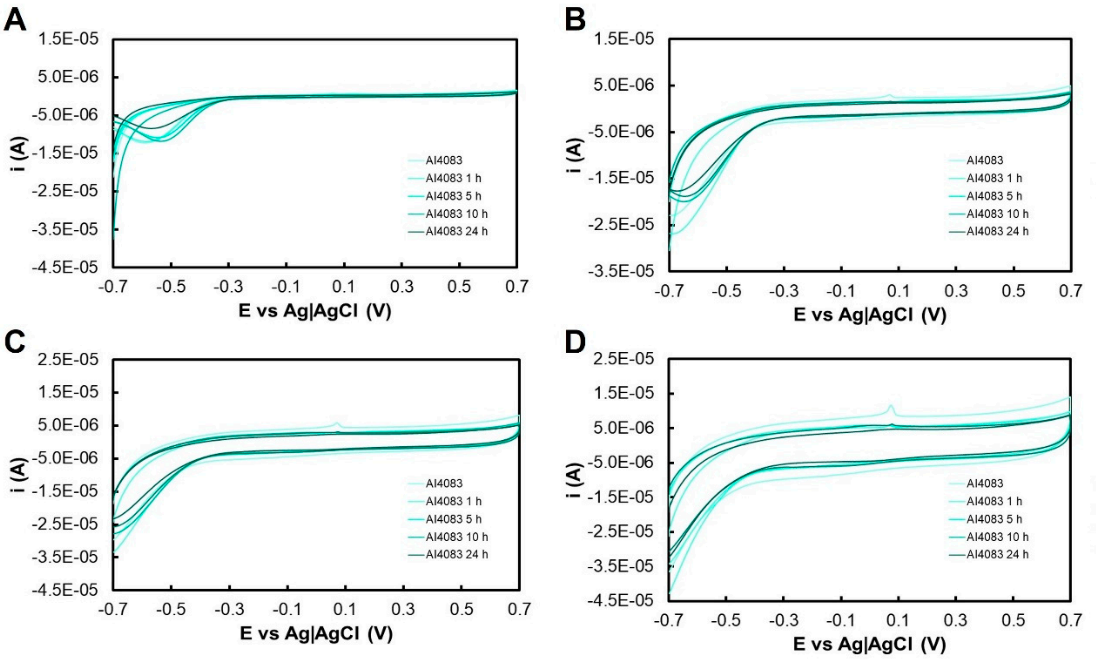

Figure S6. Cyclic voltammetry profiles of samples produced using PAN blended with PEDOT:PSS Clevios™ P VP AI 4083. Different scanning speeds were tested: (**A**) 0.01 Vs, (**B**) 0.05 Vs, (**C**) 0.1 Vs, (**D**) 0.2 Vs.

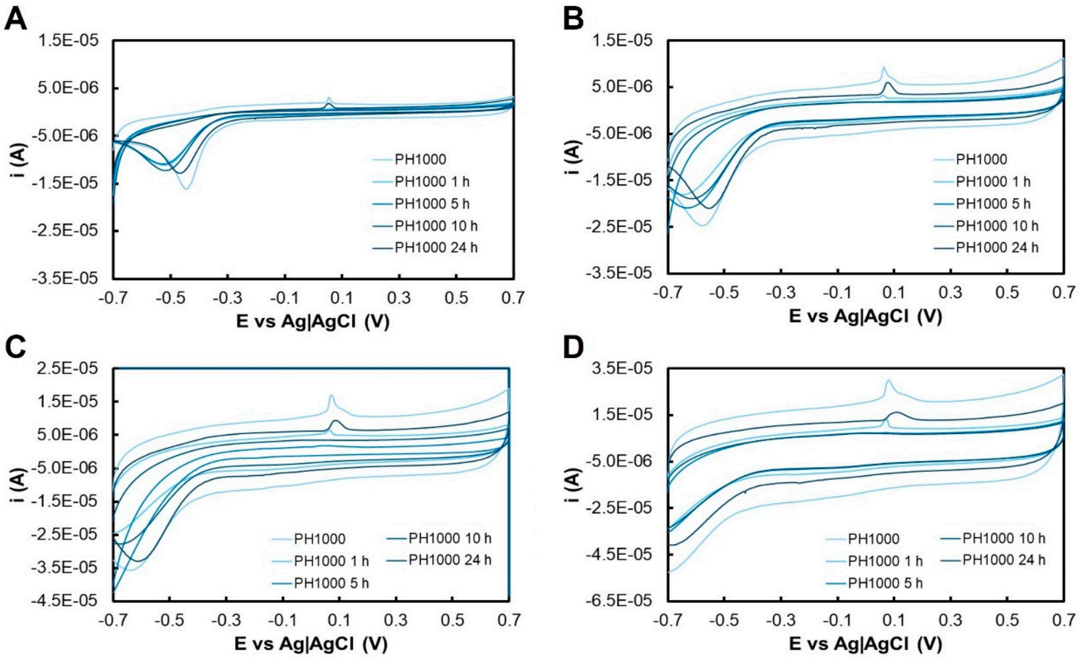

Figure S7. Cyclic voltammetry profiles of samples produced using PAN blended with PEDOT:PSS Clevios™ PH 1000. Different scanning speeds were tested: (**A**) 0.01 Vs, (**B**) 0.05 Vs, (**C**) 0.1 Vs, (**D**) 0.2 Vs.

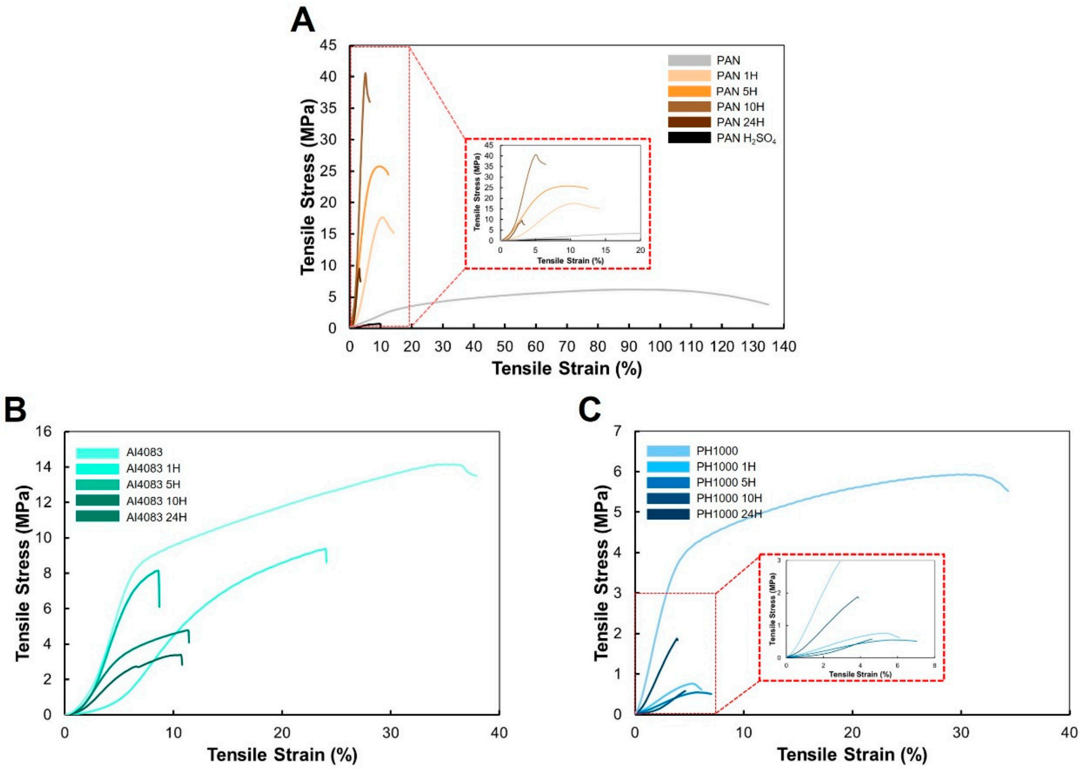

Figure S8. Examples of stress-strain curves for all samples obtained from (**A**) pristine PAN and blended with (**B**) PEDOT:PSS Clevios™ P VP AI 4083 or (**C**) PEDOT:PSS Clevios™ PH 1000.

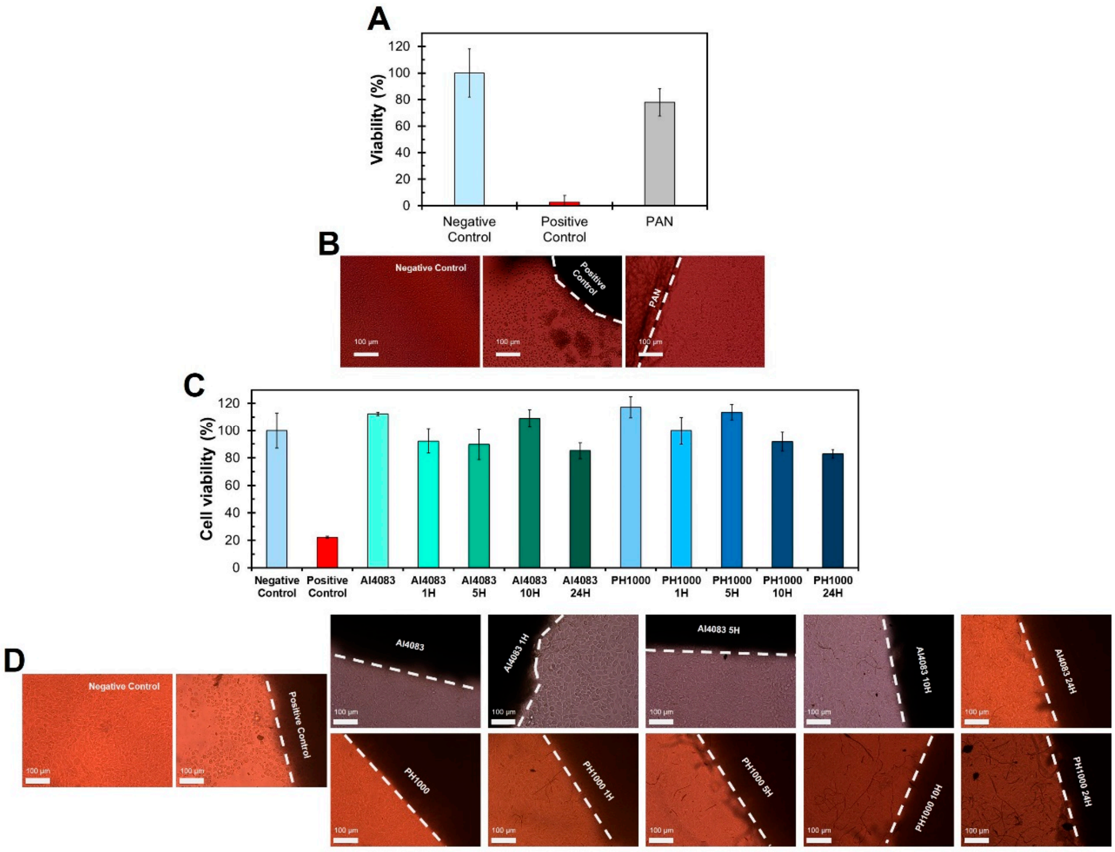

Figure S9. Summary of fiber biocompatibility evaluation following the ISO10993 guidelines for pristine PAN fibers, including (**A**) MTT after 48h of cell incubation with the lixiviates (mean ± std, n = 3) and (**B**) microscopy images depicting the direct contact results (48h); and for all the PAN/PEDOT samples obtained in this study, including (**C**) MTT after 48h of cell incubation with the lixiviates (mean ± std, n = 3) and (**D**) microscopy images depicting the direct contact results (48h).

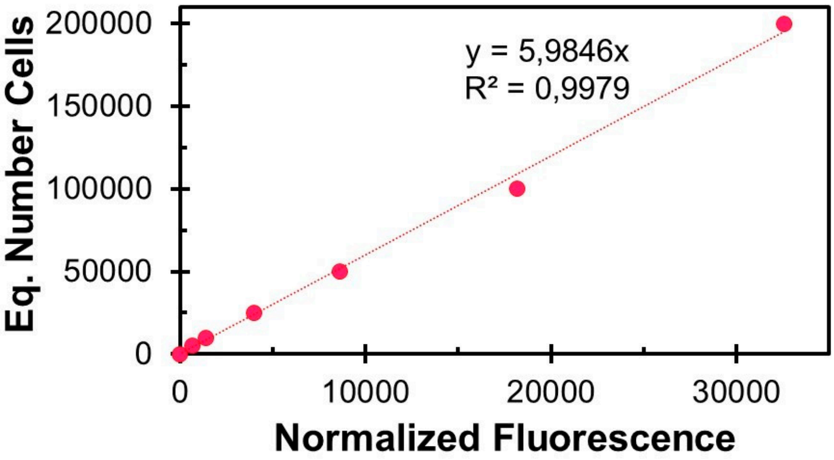

Figure S10. Alamar Blue® calibration curve for ReNCell-VM and used to calculate the equivalent number of cells in the proliferation assay.

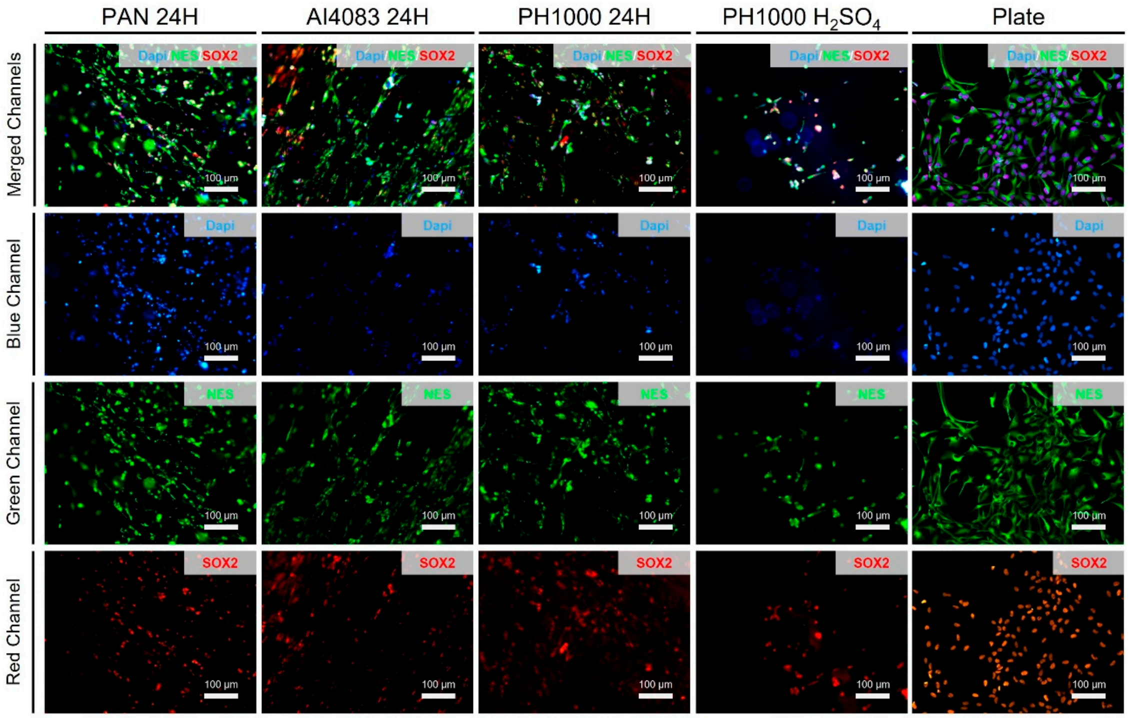

Figure S11. Immunostaining (split channels and respective merge) of ReNCell-VM after proliferating for 7 days on different samples: SOX2 (red - stem cell marker), NES (green - neural stem cell marker) and Dapi counter-stain (blue – Nucleus).

Table S1. Summary of the sample code and respective average fiber diameter (n = 50-100) of all the samples obtained in this study. (*****) means $p < 0.05$ when compared with PAN. (**+**) means $p < 0.05$ when compared with PAN 1H. (**a**) means $p < 0.05$ when compared with PAN 5H. (**b**) means $p < 0.05$ when compared with PAN 10H. (**c**) means $p < 0.05$ when compared with PAN 24H. (**d**) means $p < 0.05$ when compared with PAN H_2SO_4. (**e**) means $p < 0.05$ when compared with AI4083. (**f**) means $p < 0.05$ when compared with AI4083 1H. (**g**) means $p < 0.05$ when compared with AI4083 5H. (**h**) means $p < 0.05$ when compared with AI4083 10H. (**i**) means $p < 0.05$ when compared with AI4083 24H. (**j**) means $p < 0.05$ when compared with AI4083 H_2SO_4. (**k**) means $p < 0.05$ when compared with PH1000. (**l**) means $p < 0.05$ when compared with PH1000 1H. (**m**) means $p < 0.05$ when compared with PH1000 5H. (**n**) means $p < 0.05$ when compared with PH1000 10H. (**o**) means $p < 0.05$ when compared with PH1000 24H.

**Sample description**	**Sample code**	**Fiber Diameter (nm)**
PAN pristine	PAN	640 ± 159
PAN 1h	PAN 1H	759 ± 98$^{(*)}$
PAN 5h	PAN 5H	654 ± 135$^{(+)}$
PAN 10h	PAN 10H	703 ± 85
PAN 24h	PAN 24H	1100 ± 135$^{(*)(+)(a)(b)}$
PAN 24h + H_2SO_4	PAN H_2SO_4	612 ± 107$^{(+)(b)(c)}$
PAN/PEDOT AI4083 pristine	AI4083	356 ± 104$^{(*)(+)(a)(b)(c)(d)}$
PAN/PEDOT AI4083 1h	AI4083 1H	380 ± 108$^{(*)(+)(a)(b)(c)(d)}$
PAN/PEDOT AI4083 5h	AI4083 5H	288 ± 54$^{(*)(+)(a)(b)(c)(d)(e)(f)}$
PAN/PEDOT AI4083 10h	AI4083 10H	321 ± 95$^{(*)(+)(a)(b)(c)(d)}$
PAN/PEDOT AI4083 24h	AI4083 24H	592 ± 117$^{(*)(+)(b)(c)(e)(f)(g)(h)}$
PAN/PEDOT AI4083 24h + H_2SO_4	AI4083 H_2SO_4	507 ± 125$^{(*)(+)(a)(b)(c)(d)(e)(f)(g)(h)(i)}$
PAN/PEDOT PH1000 pristine	PH1000	515 ± 120$^{(*)(+)(a)(b)(c)(d)(e)(f)(g)(h)}$

PAN/PEDOT PH1000 1h	PH1000 1H	873 ± 179 (*)(+)(a)(b)(c)(d)(e)(f)(g)(h)(i)(j)(k)
PAN/PEDOT PH1000 5h	PH1000 5H	852 ± 210 (*)(a)(b)(c)(d)(e)(f)(g)(h)(i)(j)(k)
PAN/PEDOT PH1000 10h	PH1000 10H	685 ± 171 (a)(c)(d)(e)(f)(g)(h)(i)(j)(k)(l)(m)
PAN/PEDOT PH1000 24h	PH1000 24H	437 ± 109 (*)(+)(a)(b)(c)(d)(e)(f)(g)(h)(i)(j)(k)(l)(m)(n)
PAN/PEDOT PH1000 24h + H_2SO_4	PH1000 H_2SO_4	940 ± 210 (*)(+)(a)(b)(c)(d)(e)(g)(h)(i)(j)(k)(m)(n)(o)
